# Protein–protein interaction network-based integration of GWAS and functional data for blood pressure regulation analysis

**DOI:** 10.1186/s40246-023-00565-6

**Published:** 2024-02-08

**Authors:** Evridiki-Pandora G. Tsare, Maria I. Klapa, Nicholas K. Moschonas

**Affiliations:** 1 Department of General Biology, School of Medicine, University of Patras, Patras, Greece; 2 Metabolic Engineering and Systems Biology Laboratory, Institute of Chemical Engineering Sciences, Foundation for Research and Technology-Hellas (FORTH/ICE-HT), Patras, Greece

**Keywords:** Blood pressure regulation, Hypertension, GWAS, Human protein–protein interactions (PPIs), PPI network analysis, Network medicine, Systems medicine, Gene prioritization, Pathway enrichment analysis, Multifactorial diseases

## Abstract

**Background:**

It is valuable to analyze the genome-wide association studies (GWAS) data for a complex disease phenotype in the context of the protein–protein interaction (PPI) network, as the related pathophysiology results from the function of interacting polyprotein pathways. The analysis may include the design and curation of a phenotype-specific GWAS meta-database incorporating genotypic and eQTL data linking to PPI and other biological datasets, and the development of systematic workflows for PPI network-based data integration toward protein and pathway prioritization. Here, we pursued this analysis for blood pressure (BP) regulation.

**Methods:**

The relational scheme of the implemented in Microsoft SQL Server BP-GWAS meta-database enabled the combined storage of: GWAS data and attributes mined from GWAS Catalog and the literature, Ensembl-defined SNP-transcript associations, and GTEx eQTL data. The BP-protein interactome was reconstructed from the PICKLE PPI meta-database, extending the GWAS-deduced network with the shortest paths connecting all GWAS-proteins into one component. The shortest-path intermediates were considered as BP-related. For protein prioritization, we combined a new integrated GWAS-based scoring scheme with two network-based criteria: one considering the protein role in the reconstructed by shortest-path (RbSP) interactome and one novel promoting the common neighbors of GWAS-prioritized proteins. Prioritized proteins were ranked by the number of satisfied criteria.

**Results:**

The meta-database includes 6687 variants linked with 1167 BP-associated protein-coding genes. The GWAS-deduced PPI network includes 1065 proteins, with 672 forming a connected component. The RbSP interactome contains 1443 additional, network-deduced proteins and indicated that essentially all BP-GWAS proteins are at most second neighbors. The prioritized BP-protein set was derived from the union of the most BP-significant by any of the GWAS-based or the network-based criteria. It included 335 proteins, with ~ 2/3 deduced from the BP PPI network extension and 126 prioritized by at least two criteria. ESR1 was the only protein satisfying all three criteria, followed in the top-10 by INSR, PTN11, CDK6, CSK, NOS3, SH2B3, ATP2B1, FES and FINC, satisfying two. Pathway analysis of the RbSP interactome revealed numerous bioprocesses, which are indeed functionally supported as BP-associated, extending our understanding about BP regulation.

**Conclusions:**

The implemented workflow could be used for other multifactorial diseases.

**Supplementary Information:**

The online version contains supplementary material available at 10.1186/s40246-023-00565-6.

## Background

For more than a decade now, genome-wide associations studies (GWAS) have been an important method in genomic analysis, furthering our knowledge of the genetic basis of complex phenotypes through the identification of hundreds to thousands associated genetic variants [1]. GWAS data are usually augmented by expression quantitative trait loci (eQTL) measurements, which identify variant-gene associations based on changes in gene expression [2]. Over the years, few publicly available general repositories of GWAS data over various phenotypes have been developed, with GWAS Catalog being the most prominent resource as a collaborative effort of EMBL-EBI and NHGRI [3]. However, there is a lack of specialized GWAS meta-databases for specific diseases or complex phenotypes, developed based on a systematic mining of the large repositories and the literature over the associated traits, to provide a comprehensive resource for exploring the currently known genetic basis of the particular disease or phenotype. To date, new GWAS publications present mainly the newly identified loci for a particular phenotype, and there are a limited number of reported meta-analyses over phenotype-associated variants or genes reported in multiple studies. Comprehensive specialized GWAS data collections will enable the prioritization of disease-related genes based on an extended set of criteria, including, apart from the associated *p*-value, the number of supporting studies and the number of identified variants per gene locus among others. Furthermore, disease-specific GWAS meta-databases connecting GWAS with biological data will enable the direct integration of the GWAS measurements into high-throughput molecular phenotyping analyses of the particular pathophysiology [4].

Collecting and analyzing the full GWAS dataset for a particular complex phenotype is very important, as the specific physiology results from the combined inter-regulation of multiple interacting polygenic pathways, rather than the isolated effect of certain genes [5–7]. Hence, considering the GWAS-identified disease-related genes individually may explain only a small portion of the underlying molecular mechanisms of the specific pathophysiology [8]. It is of value to upgrade the information content of GWAS data through their analysis in the context of biomolecular interaction networks [9]. Proteins being the main functional and regulatory biomolecules, usually operating in interacting modules, the protein–protein interaction networks provide a reliable representation of the interconnectivity between molecular functions [10, 11]. In this context, protein–protein interaction (PPI) networks have been widely used in network medicine for the investigation of the molecular architecture of diseases and pathophysiologies through the collective analysis of genomic data [12–14]. Reconstructing the disease-associated protein interactome by reflecting comprehensive disease-specific GWAS data collections over the human PPI network provides a wider perspective of the involved molecular pathways, the combined deregulation of which could lead to disease conditions. Prioritization of proteins based on their position and role in the reconstructed disease-associated network can lead to more reliable risk factor indicators [15–17]. Furthermore, analysis of the GWAS-based network could direct to the identification of newly implicated genes through the underlying assumption of “guilt-by-association” principle [18, 19], according to which genes involved in the same biological processes are more likely to be associated with the same or similar phenotypes.

Derailment of blood pressure (BP) regulation is associated with a vast number of pathophysiologies, including heart failure and cardiovascular diseases, stroke and renal failure [20]. Elucidating its genetic basis would have a positive effect in the development of diagnostic tools, effective therapeutic treatments and new drugs in a spectrum of diseases that affect a large portion of the human population. Many GWAS focusing on BP traits have been performed since 2007 and have revealed a high number of associated genetic loci [21–24]. However, no BP-specific GWAS database currently exists, collecting all this information into one resource. BP-GWAS data have been analyzed in the context of PPI networks [25–27], but these studies are mainly based on one or few GWAS. A comprehensive BP-specific GWAS data resource, augmented with eQTL measurements and involving information of variant-gene associations, could be a very useful tool in systems and network biology investigations to understand BP regulation and related dysfunctions.

To this end, we aimed at developing a systematically literature-curated BP-GWAS meta-database, augmented with cis-eQTL and gene-variant association data. Using the included information in an integrated way, extended GWAS-based prioritization criteria for disease-associated genes can be considered. A second major objective of the study was to reconstruct the BP-associated PPI network considering the comprehensive GWAS data collection and use it to identify new BP-related genes. The extended GWAS-based network was used to identify important BP-related pathways and prioritize the proteins based on their position according to network interconnectivity metrics.

## Methods

### GWAS data

Τhe GWAS Catalog database (https://www.ebi.ac.uk/gwas/) [3] was used as the main resource to retrieve BP-associated GWAS data, mining the spreadsheet files “All Associations”, “All studies” and “All ancestry data”. It is noted that for each single nucleotide polymorphism (SNP), GWAS Catalog records mainly the most significant (i.e., with the lowest *p*-value) SNP-trait association from a particular study. Thus, for multi-stage studies, it may focus on the combined stage, skipping significant association *p*-values at other stages. In the same context, for BP multi-trait studies, only the trait with the most significant association *p*-value is usually reported for a particular SNP. Thus, we proceeded to manually curate many large-study publications retrieved from GWAS Catalog along with some not reported in this repository at the time of BP-GWAS data collection, to extend the collected information from the literature. More specifically, for the manually curated publications, (i) we collected all significant SNP—BP trait associations at each of the initial, replication and combined stages of multi-stage studies, (ii) we recorded all BP traits significantly associated with a particular SNP at any of the study stages and the corresponding *p*-values, and (iii) we collected all significant SNPs for each reported independent locus. The Manhattan plot for the BP-GWAS meta-dataset was visualized using the R package qqman [28].

### SNP genotypic information

SNP genotypic information was collected from Ensembl using the BioMart software suite [29]. The severity of the SNP-transcript consequences (GWAS-transcripts) was recorded as defined in Sequence Ontology (http://www.sequenceontology.org/) [30] and reported in Ensembl Variation database. The corresponding genes of the GWAS-transcripts (to be called GWAS-genes) were retrieved from Ensembl. In the rest of the text, BP-genes will be referred to by their gene symbol. The chromosome map for the recorded BP-associated SNPs was visualized using the PhenoGram software tool [31].

### eQTL data

Significant tissue-specific SNP-gene associations (*q*-value ≤ 0.05) based on cis-eQTL measurements were collected from the Genotype-Tissue Expression Portal (GTEx) v.8 (https://gtexportal.org) [32, 33].

### Human PPI network: PICKLE meta-database

The human PPI network was retrieved from the Protein InteraCtion KnowLedgebasE (PICKLE) (www.pickle.gr) [10, 11, 34, 35]. The unique feature of PICKLE is that primary datasets of experimental PPIs are integrated on the genetic information ontology network of the UniProt/SwissProt-defined reviewed human complete proteome (RHCP) (https://www.uniprot.org/) [36], without a priori transformations to a pre-selected genetic information level. The PICKLE ontology network includes the associations between the RHCP UniProt IDs and their encoding genes and transcripts [34]. PICKLE reports three versions of the human PPI network, i.e., unfiltered, standard, cross-checked (default), with increasing experimental reliability for the involved PPIs of being direct. In this study, we used mainly the default version, mentioning the cases where investigated PPIs or UniProt IDs are involved only in other versions. In the rest of the text, proteins will be referred to by their UniProt Entry Name (excluding the extension _HUMAN).

### Network visualization and analysis

PPI network visualization was carried out using Cytoscape version 3.7.2 (https://cytoscape.org/) [37]. Network analysis was carried out with the relevant Cytoscape plugin. The role of the nodes in a PPI network was also evaluated based on the “Integrated Value of Influence (IVI)” metric [38]. IVI combines six topological features of a node as follows:

1 $${\text{IVI}}_{i} = \left( {{\text{DC}}_{i}^{\prime } + {\text{LH}}_{{{\text{index}}_{i} }}^{\prime } } \right) \left( {\left( {{\text{NC}}_{i}^{\prime } + {\text{CR}}_{i}^{\prime } } \right) \left( {{\text{BC}}_{i}^{\prime } + {\text{CI}}_{i}^{\prime } } \right)} \right)$$

where ${\text{DC}}_{i}^{\prime }$, ${\text{LH}}_{{{\text{index}}_{i} }}^{\prime }$, ${\text{NC}}_{i}^{\prime }$, ${\text{CR}}_{i}^{\prime }$, ${\text{BC}}_{i}^{\prime }$, ${\text{CI}}_{i}^{\prime }$ are, respectively, the [1–100] min–max range-normalized, degree centrality [39], local H index [40], neighborhood connectivity [41], ClusterRank [42], betweenness centrality [39] and collective influence [43], of node i. The sum in the first parenthesis, $\left( {{\text{DC}}_{i}^{\prime } + {\text{ LH}}_{{{\text{index}}_{i} }}^{\prime } } \right)$, is a measure of the node “hubness”, depending on the number of the node interactions and the semi-local centrality measure, LH index. The complex product in the second parenthesis, $\left( {\left( {{\text{NC}}_{i}^{\prime } + {\text{CR}}_{i}^{\prime } } \right) \left( {{\text{BC}}_{i}^{\prime } + {\text{CI}}_{i}^{\prime } } \right)} \right)$, is a measure of the node “spreading”. Τhe first part of the spreading measure, $\left( {{\text{NC}}_{i}^{\prime } + {\text{CR}}_{i}^{\prime } } \right)$, combines the semi-local and local, respectively, centrality measures, neighborhood connectivity and ClusterRank, and may reveal semi-local hubs of the network too. Τhe second part, $\left( {{\text{BC}}_{i}^{\prime } + {\text{CI}}_{i}^{\prime } } \right),$ reveals proteins important in maintaining the connectivity of the network.

*Gene/Protein Prioritization Threshold*: In all cases of gene/protein scores or metrics, including IVI, unless otherwise specified, the used threshold of significance for gene/protein prioritization is as follows:

2 $${\text{Significance}}\_{\text{threshold}} = {\text{mean}}\left( {{\text{Score}}_{i = 1, \ldots, \text{N}} } \right) + 1.5 {\text{std}}\left( {{\text{Score}}_{i = 1,\ldots,\text{N}} } \right)$$

where ${\text{mean}}\left( {{\text{Score}}_{i = 1,\ldots,\text{N}} } \right)$ and ${\text{std}}\left( {{\text{Score}}_{i = 1,\ldots,\text{N}} } \right)$ are, respectively, the mean and standard deviation of the ${\text{Scores}}\;{\text{ or}} \, {\text{Metric}}\,\,{\text{values}}$ of all (N) considered genes/proteins and most significant are considered the genes/proteins with ${\text{Score or}} \,{\text{Metric}}\,\,{\text{value}} \ge {\text{Significance}}\_{\text{threshold}}.$

### Pathway enrichment analysis

Pathway enrichment analysis was performed using the Database for Annotation, Visualization and Integrated Discovery (DAVID) version 6.8 (https://david.ncifcrf.gov/) [44, 45] and the pathway maps of Kyoto Encyclopedia of Genes and Genomes (KEGG) release 92.0 (https://www.kegg.jp/), through KEGG mapper [46–48].

### Drug–protein and gene–disease associations

Antihypertensive drugs targeting BP-proteins were mined from UniProtKB (as curated in PICKLE) and DrugBank version 5.1.4 (https://www.drugbank.ca/) [49]. In the latter, we searched for drug descriptions that contained at least one of the “hyperten-” or “blood pressure reduction” text strings. OMIM (https://www.omim.org/) [50], UniProtKB and GAD (as curated in DAVID) [51] databases were used to retrieve gene–disease associations for the BP-proteins.

## Results

**Fig. 1:**
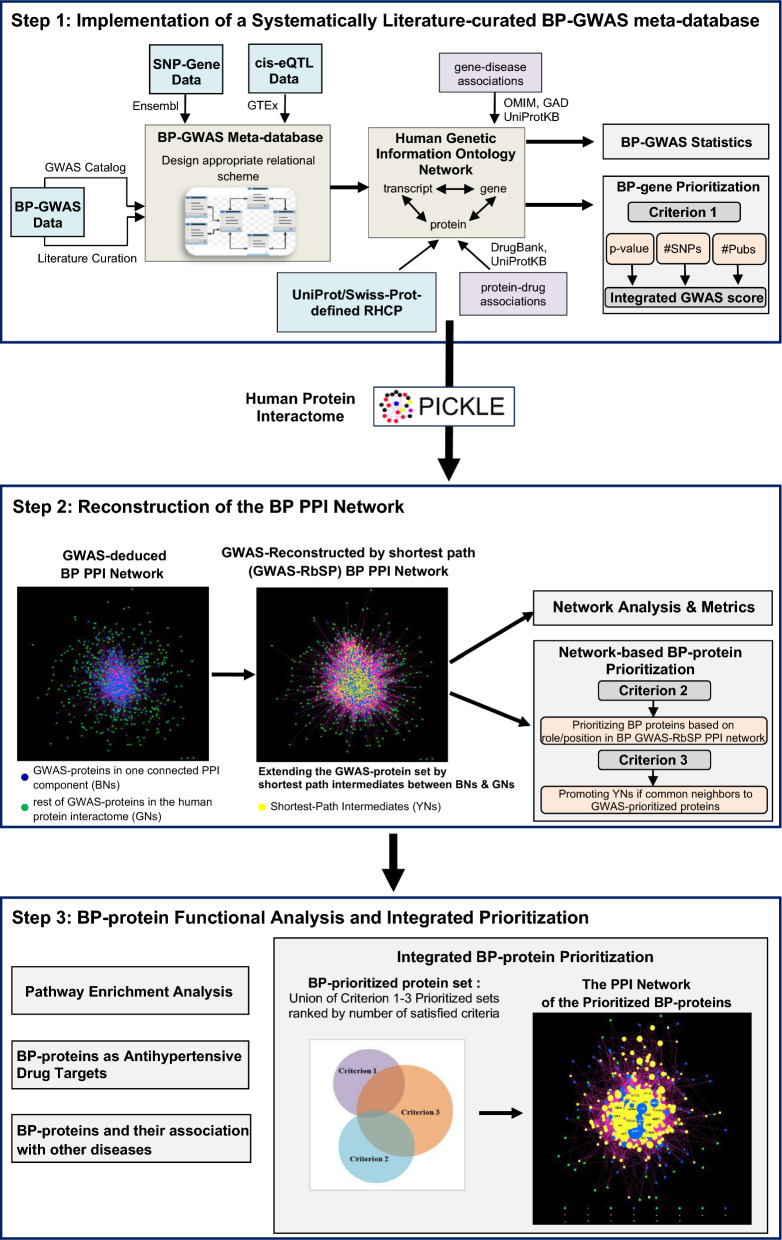
The workflow of the integrated BP-GWAS data and PPI network analyses

The workflow followed in this study, as shown in Fig. 1, includes: (1) the implementation of a systematically literature-curated BP-GWAS meta-database enriched with SNP-transcript associations and eQTL data, which was linked with the RHCP genetic information ontology network of PICKLE augmented by gene–disease and drug–protein association data, (2) the reconstruction of the BP-associated PPI network using PICKLE, from the interactions between the proteins encoded by the GWAS-genes (to be called as GWAS-proteins), extended by the shortest interaction paths connecting all GWAS proteins into one component, and (3) the PPI network-based integration of the GWAS and the functional data for pathway enrichment analysis and protein prioritization. The latter was accomplished by a newly proposed integration of a GWAS-based and two network-based criteria. The various steps of the workflow are described in detail below.

### Implementation of the BP-GWAS meta-database

#### Relational scheme

**Fig. 2:**
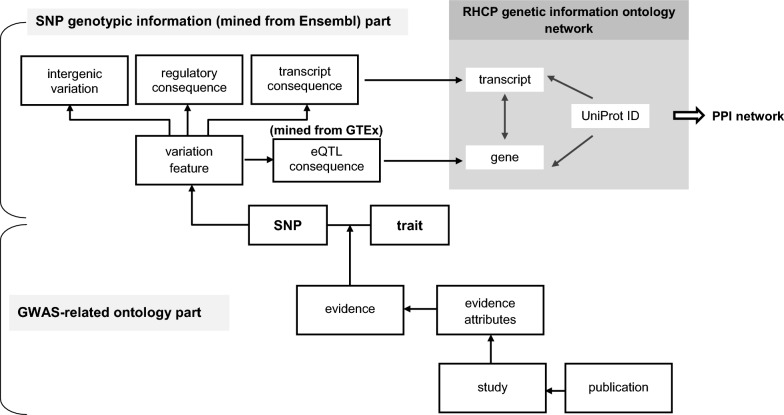
The relational scheme of the BP-GWAS meta-database. The scheme comprises two parts: the GWAS-related ontology part (bottom) and the SNP genotypic information part (top). The meta-database is connected to the RHCP-based genetic information ontology network of the PICKLE PPI meta-database at the transcript and gene levels, through SNP–transcript consequences and eQTL measurements, respectively

The meta-database was designed as shown in Fig. 2, to systematically store BP-GWAS data and their attributes (GWAS-related ontology part), and include SNP-transcript associations and eQTL data (SNP genotypic information part). The SNP–transcript associations link the GWAS data to the genetic information ontology network connecting genes, transcripts and proteins, and thus consequently, to any type of biological, omic and functional data, including drug–protein associations, gene–disease associations and PPIs. In more detail, the two meta-database parts are structured as follows:

##### GWAS-related ontology part

Each recorded SNP–trait association *p*-value is related to the study in which the association was identified and the publication reporting the study. In our ontology, each independent study is uniquely defined by a profile of attributes adapted from the GWAS Catalog data files, excluding thus any ambiguity about the study that revealed a SNP–trait association in the case of multi-study publications. Furthermore, the GWAS meta-database can be queried for any combination of study attributes to identify more specific BP-GWAS data subsets. The unique study attribute profile comprises: (a) the analysis stage (i.e., initial, replication or combined); (b) the number of involved samples; (c) the ancestry profile of the involved individuals based on the GWAS Catalog-defined ancestry categories [52]; (d) the broader ancestry and the number of the concerned individuals; (e) the country/ies of the individuals’ recruitment; (f) the country/ies of the individuals’ origin (if available); (g) the type of study (GWA or exome array study); (h) the genotyping array used; (h) the gender and age of the individuals (if any or both are specified in specialized studies), and (i) the statistical measure based on which the SNP–trait association *p*-values are calculated.

##### SNP genotypic information part

It is structured based on the relevant part of the Ensembl variation database scheme, and the information stored for each GWAS-identified SNP includes the chromosomal location, the minor allele, the global minor allele frequency and the transcript, regulatory and motif consequences. The transcript consequences of a SNP (GWAS-transcripts) are assigned an Ensembl-defined severity score, using a scale from 1 (most severe— “transcript_ablation”) to 35 (least severe—“feature_truncation”). Intergenic SNPs, considered of the lowest severity, are recorded separately. In this way, our meta-database can store all transcript consequences for the GWAS-identified SNPs, independently of their severity. This is a distinctive feature over other GWAS data collections, usually storing only the most severe transcript consequence per SΝP. Recording all GWAS-transcripts enlarges the perspective of the BP-associated molecular physiology that can be extracted from GWAS. It also provides the ability for the user to only select the BP-associated transcripts above a particular severity threshold and investigate any potential variations in the derived information about the investigated phenotype. Finally, our meta-database stores locally eQTL information for the GWAS-identified SNP-gene associations.

Importantly, as structured, the proposed meta-database scheme is not specific to BP, but it is applicable to GWAS data collections of any multifactorial disease.

#### Populating the meta-database with BP-GWAS data

The GWAS-related part of the meta-database was populated with the SNP-BP trait associations with *p*-value < 10^−5^ and their attributes, as mined from: (i) the GWAS Catalog and (ii) manually curated BP-GWAS publications. GWAS Catalog “mapped” traits were identified as BP-related (Additional file 1: Table S1), if comprising at least one BP-associated Experimental Factor Ontology term (EFO, https://www.ebi.ac.uk/efo/) (Additional file 1: Table S2). Then, we mined all information from the 69 identified as BP-related publications as stored in GWAS Catalog, and proceeded to manually curate 22 more recent and larger of them and one additional publication not at the time curated by GWAS Catalog (Additional file 1: Table S3), as described in Methods. In the eQTL measurement section, we mined from GTEx any significant cis-eQTL association *q*-values, i.e. < 0.05, for the GWAS-identified SNP-gene pairs in tissues and their regions (27 terms in total), considered to be involved in BP regulation: the heart, artery, whole blood, kidney, adipose tissue, brain, adrenal gland, thyroid, skeletal muscle, liver, and tibial nerve. The eQTL-significant genes (by any of the GWAS-identified SNPs) in a particular tissue or tissue region will be referred to as eGenes in this tissue or tissue region.

The stored GWAS data were connected to the human genetic information ontology network via the GWAS-transcripts (Fig. 2). The corresponding GWAS-genes were connected to diseases through relevant databases, and the PPI network reconstruction was based on the encoded GWAS-proteins, which were also investigated as drug targets (Fig. 1). The RHCP-included GWAS-proteins in the PICKLE PPI database ontology network were used to reconstruct the GWAS-deduced BP-protein interactome. The eQTL data were integrated with the rest through the eGenes.

#### Statistics of the BP-GWAS meta-database

##### At the SNP level

The systematic curation of 70 BP-GWAS research papers published since 2007 (Additional file 1: Table S3), involving more than 1.5 million samples from 14 ancestries and 212 independent studies, led to the collection of 7401 SNPs associated with BP trait(s) with *p*-value < 10^−5^ (Table 1). The collected SNP-BP trait association *p*-values sum up to 27,480, 98% of which are reported in the after 2016 publications (Additional file 1: Table S3). Notably, 95% of the *p*-values are from the 23 manually curated publications. If the stricter, presently used, 5 × 10^−8^ *p*-value significance threshold is considered, our dataset comprises 21,788 SNP−BP trait association *p*-values for 6687 SNPs. These data were acquired in 151 independent studies involving samples from 13 ancestries, reported in 54 publications (Table 1, Additional file 1: Table S3). In this narrower GWAS dataset, which will be used in the rest of the analysis as the significantly BP-associated, 98% of the *p*-values were retrieved from the manually curated references. This indicates that the vast majority of the stored and analyzed BP-GWAS data are based on our systematic and extended mining of the BP-GWAS literature.

**Table 1:** The size of the curated BP-GWAS dataset at two significant SNP–trait association *p*-value thresholds

	*p*-value < 10^−5^	*p*-value < 10^−8^
SNPs	7401	6687
SNP–trait associations	27,480	21,788
Publications	70	54
Independent studies	212	151

The 6687 BP-associated SNPs are distributed in all 22 human autosomes with only two (rs141216986, rs6609273) in chromosome X and none in chromosome Y (Additional file 1: Table S4). The vast majority of the SNPs are associated with at least one of the systolic or diastolic or pulse pressure measurement traits (6591) and are supported by at most two publications (5688 by one and 768 by two). Four SNPs were reported in 11 publications, i.e., rs17249754 (*ATP2B1*, 16 studies), rs11191548 (*CNNM2*, 14 studies), rs3184504 (*SH2B3*, 13 studies) and rs1458038 (intergenic, 19 studies), and two SNPs in 10 publications, i.e., rs880315 (*CASZ1*, 16 studies), rs13107325 (*SLC39A8*, 13 studies). The two SNPs supported by the maximum number (21) of independent studies, i.e., rs167479 (*RGL3*) and rs16998073 (intergenic), were reported in 6 and 9 publications, respectively.

**Fig. 3:**
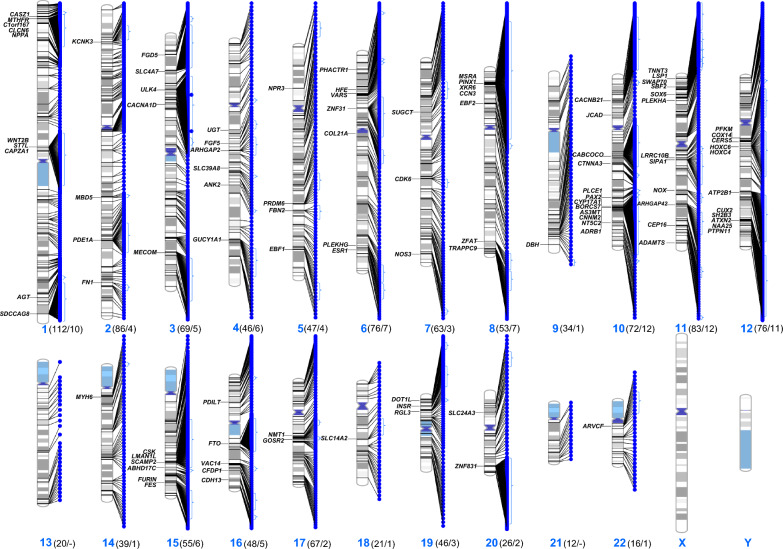
Chromosome map of the BP-SNPs indicating the GWAS-prioritized RHCP-coding gene sites. The numbers of all and the GWAS-prioritized (in parenthesis) RHCP-coding BP-genes of each human chromosome are also shown

**Fig. 4:**
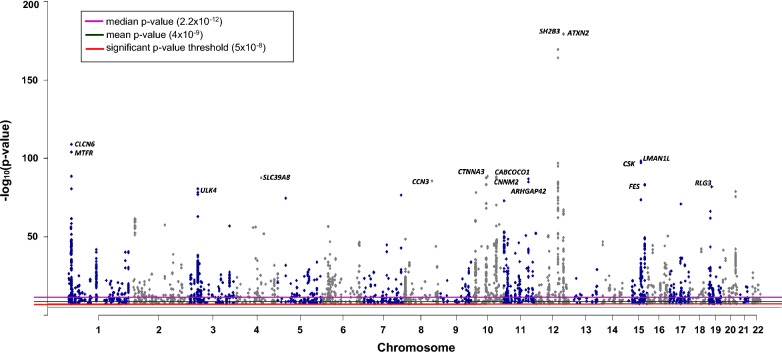
Manhattan plot of the RHCP-coding SNPs based on their minimum BP-association *p*-value. The red, black and purple lines denote, respectively, the presently used 5 × 10^−8^ *p*-value threshold for significant SNP-trait associations, the mean, and the median minimum BP-association *p*-value of the GWAS-identified SNPs. The genes with the smallest BP-association *p*-values are also shown

**Fig. 5:**
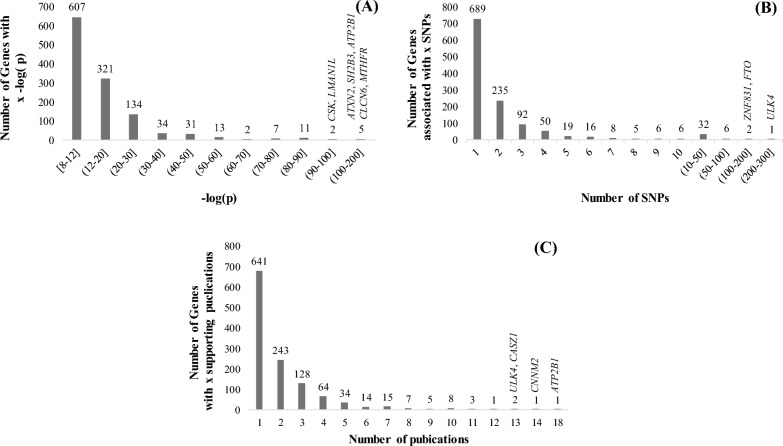
RHCP-coding BP-gene distributions by minimum *p*-value (**A**), SNP number (**B**), supporting publication number (**C**)

About 56% (3738) of the significant SNPs have RHCP-coding transcript consequences, while ~ 16% have only non-coding transcript consequence(s) and ~ 24% are intergenic (Additional file 1: Table S5). The 3738 SNPs are associated with 1167 RHCP-coding genes (Fig. 3; Additional file 1: Table S5; complete list in Additional file 2). The median of the minimum BP-association *p*-values of the RHCP-related SNPs is 2.2 × 10^−12^ (Fig. 4). Half (585) of the 1167 RHCP-coding genes are associated with SNPs of minimum BP-association *p*-value smaller than the median (Fig. 5A, Additional file 2). This observation may indicate the 2.2 × 10^−12^ *p*-value as a new stricter genome-wide significance threshold for SNP-BP trait associations identified in GWAS, compared to the current generally considered 5 × 10^−8^ value. Any further analysis will refer to the BP-associated RHCP-coding genes or transcripts.

##### At the protein-coding gene level

Among the RHCP-coding BP genes, ~ 41% are related with at least two significant SNPs, ~ 9% (101) with at least five and nine genes with more than fifty SNPs (Fig. 5B, Additional file 2). These 9 genes are: *ULK4* (Chromosome 3, 276 SNPs), Z*NF831* (Chromosome 20, 124 SNPs), *FTO* (Chromosome 16, 103 SNPs), *SLC4A7* (Chromosome 3, 85 SNPs), *MSRA* (Chromosome 8, 82 SNPs), *CLCN6* (Chromosome 1, 79 SNPs), *PINX1* (Chromosome 8, 68 SNPs), *CNNM2* (Chromosome 10, 59 SNPs) and *CABCOCO1* (Chromosome 10, 58 SNPs). For 9% (105) of the genes, the minimum BP-association *p*-value is smaller than 10^−30^, and for 18 genes it is smaller than 10^−80^ (Fig. 5A, Additional file 2). The smallest *p*-values encountered in our dataset correspond to SNPs associated with Ataxin 2 (*ATXN2*, *p*-value = 4.8 × 10^−180^) and SH2B adapter protein 3 (*SH2B3*, *p*-value = 8 × 10^−180^), both mapped on chromosome 12 (Fig. 4, Additional file 2). The association of ~ 45% of the RHCP-coding BP-genes is supported by at least two publications, for 13% (155) by at least four and for eight genes by more than ten independent GWAS publications (Fig. 5C, Additional file 2). These genes are: *ATP2B1* (Chromosome 12, 18 publications), *CNNM2* (14 publications), *CASZ1* (Chromosome 1, 13 publications), *ULK4* (13 publications), *ARHGAP42* (Chromosome 11, 12 publications), *SH2B3* (11 publications), *FES* (Chromosome 15, 11 publications) and Z*NF831* (11 publications). These observations indicate that while the vast majority of the BP-associated SNPs have been reported in a single publication, considering all significant SNPs per gene increases the reliability of the BP-association at the gene level as this is now supported by multiple publications and multiple SNPs.

**Fig. 6:**
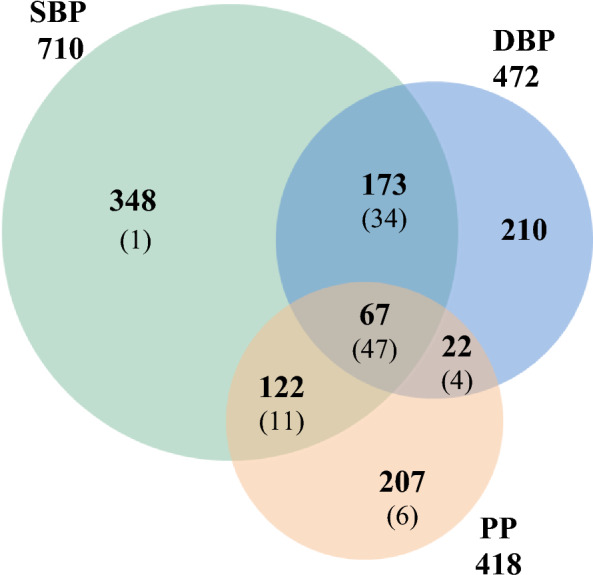
Venn diagram of the RHCP-coding BP-genes associated with systolic, diastolic and pulse pressure traits. SBP, DBP and PP depict, respectively, the systolic blood pressure, the diastolic blood pressure and the pulse pressure traits. The respective gene numbers in the GWAS-prioritized set are shown in parenthesis

Overall, 56 RHCP-coding genes can be considered as the most BP-significant set based on the GWAS data, as they are in the top 10% with respect to all three GWAS attributes, i.e., they are related to at least five SNPs with a minimum *p*-value < 10^−30^ as reported in at least two publications. Chromosomes 1, 2 and 11 are the most enriched in BP-associated genes (Additional file 1: Table S4). Only 2% of the BP-associated genes are related to other BP-traits than the systolic (710), or diastolic (472), or pulse (418) pressure (Fig. 6, Additional file 2). Sixty-seven genes are associated with all three of these three traits. It needs to be noted, however, that the number of GWAS publications investigating pulse pressure is much smaller compared to those for systolic or diastolic pressure (Additional file 2).

##### eQTL measurements and tissue specificity

**Fig. 7:**
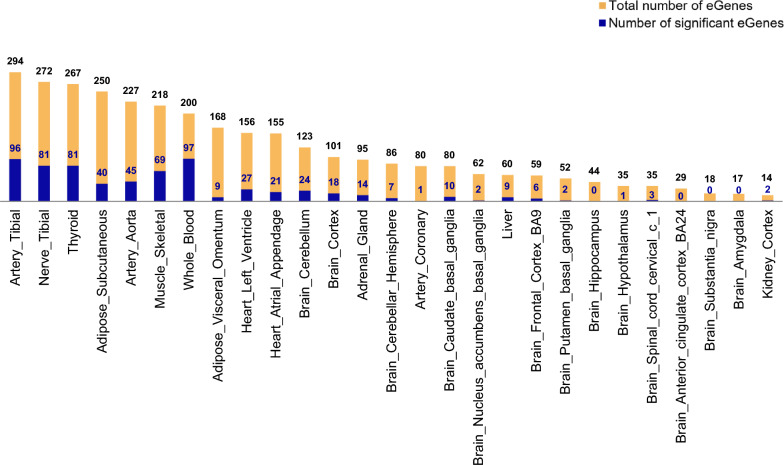
The tissue distribution of RHCP-coding BP-eGenes. The distribution is shown for the 27 selected as BP-related tissues in ascending number of eGenes per tissue. Significant eGenes for a tissue are those exhibiting in that tissue their minimum q-value among all 27 tissues

We discovered that 665 (57%) out of the 1167 RHCP-coding genes in the BP-GWAS dataset were detected as eGenes in at least one of the 27 selected as BP-related tissues (Additional file 3). Thirty-eight of them are eGenes in more than 14 tissues and one (*AMH;* Anti-Mullerian hormone) in all 27 tissues. Οn the other hand, the tissues with more than 200 eGenes are the artery tibial, the nerve tibial, the thyroid, the adipose subcutaneous, the artery aorta, the muscle skeletal and the whole blood (Fig. 7). Finally, after flagging an eGene as significant in a tissue when exhibiting in this tissue its minimum q-value over the 27 tissues, the tissues observed with the highest number of significant eGenes were the whole blood, the artery tibial, the nerve tibial, the thyroid and the skeletal muscle (Fig. 7, Additional file 3).

### Gene prioritization by integrated GWAS-based criterion

#### Integrated GWAS-based gene scoring scheme

The proposed gene scoring scheme is based on the combined consideration of three GWAS data attributes: the gene association *p*-value with the GWAS-investigated phenotype, the number of significant SNPs per gene and the number of supporting GWAS publications. Thus, we propose an integrated score for gene *i*, $s_{i}$, defined as the weighted sum of these three GWAS-attribute values, $p - {\text{value}}_{i} , \# {\text{SNPs}}_{i} , \# {\text{Pubs}}_{i,}$ [1–100] min–max range-normalized (denoted by the symbol $\widehat{{\phantom{a}}}$ in Eq. 3):

3 $$s_{i} = w_{{p - {\text{value}}}} \times \left| {\log (\widehat{{p - {\text{value}}_{i} )}}} \right| + w_{{{\text{SNP}}}} \times \widehat{{\# {\text{SNPs}}_{i} }} + w_{{{\text{Pub}}}} \times \widehat{{\# {\text{Pubs}}_{i} }}$$

$w_{{p - {\text{value}}}}$, $w_{{{\text{SNP}}}}$ and $w_{{{\text{Pub}}}}$ are the respective % weights of the three GWAS attributes.

Among the three GWAS attributes in Eq. 3, we consider the *p*-value as being the most indicative for the association of a gene with the GWAS-investigated phenotype. While the number of significant SNPs per gene is also important, we consider it of lower weight for gene prioritization, because of the current, still considerate, bias in these data. To our knowledge, until recently (2016) the researchers tended to report only the characteristic SNP per locus [21], independently of which SNP(s) had been identified as significant. The weight for the number of independent publications supporting the association of a gene/locus with the investigated phenotype may be the lowest among the three factors in the integrated score, because GWAS publications usually report only the newly identified loci and skip information about confirmed loci that have already been reported in previous publications. As observed in the BP-GWAS data too, a vast number of GWAS-identified loci/genes tend still to be supported by a single publication. In this context, our present suggestion is for the $w_{{p - {\text{value}}}}$, $w_{{{\text{SNP}}}}$ and $w_{{{\text{Pub}}}}$ to be, respectively, 45%, 35% and 20%. These relative weights may be re-evaluated in the near future, as the contribution of the above-mentioned biases diminishes with the progress of GWAS and genomic analyses.

For the prioritization of genes based on their integrated GWAS-based score, we opted for the normal distribution-based lenient significance threshold of Eq. 4:

4 $$s_{{{\text{cut}} - {\text{off}}}} = {\text{mean}}\left( {s_{i = 1, \ldots, N} } \right) + {\text{std}}\left( {s_{i = 1, \ldots, N} } \right)$$

where ${\text{mean}}\left( {s_{i = 1 ,\ldots, N}} \right)$ and ${\text{std}}\left( {s_{i = 1 ,\ldots, N}} \right)$ are, respectively, the mean and standard deviation of all gene scores. Significantly associated with the GWAS-investigated phenotype are considered the genes with scores equal to or greater than the cut-off value (Criterion 1).

#### The prioritized BP-associated gene set by the integrated GWAS-based score

The integrated GWAS-based scores for all 1167 RHCP-coding BP-associated genes are shown in Additional file 2. Based on Eq. 4 significance threshold, the Criterion 1-prioritized set includes 103 genes (~ 9%) (Table 2, Additional file 2), comprising all but one of the 56 genes identified in the top 10% for all three GWAS attributes combined in the integrated score. Namely, *ULK4* (Unc-51 Like Kinase 4, s_ULK4_ = 100) and *ATP2B1* (ATPase Plasma Membrane Ca^2+^ Transporting 1, s_ATP2B1_ = 99.5) exhibit the highest scores. Thirteen (13) genes have a score greater than 50.

**Table 2:** The prioritized BP-associated genes based on the integrated GWAS score (Criterion 1)

Rank	Gene Symbol	*p*-value	#SNPs	#Pubs	Score	Rank	Gene Symbol	*p*-value	#SNPs	#Pubs	Score	Rank	Gene Symbol	*p*-value	#SNPs	#Pubs	Score
1	***ULK4***	81	276	13	100	36	***FTO***	21	103	4	29.9	71	***CERS5***	50	1	3	20.4
2	***ATP2B1***	170	44	18	99.5	37	*ADRB1*	58	1	6	28.5	72	***CEP164***	34	6	6	20.3
3	*SH2B3*	180	5	11	84.1	38	***HOXC4***	41	26	7	28.4	73	*C1orf167*	45	10	3	20.1
4	*ATXN2*	180	22	8	82.1	39	***LRRC10B***	46	4	8	27.9	74	*MBD5*	58	1	1	20
5	*ZNF831*	79	124	11	67.8	40	***CDH13***	51	2	7	27.8	75	***ARVCF***	28	7	7	19.9
6	***CNNM2***	87	59	14	63.9	41	***WNT2B***	42	18	7	27.3	76	***ADAMTS8***	52	2	2	19.6
7	***CLCN6***	109	79	6	62.3	42	***PLCE1***	46	8	7	27	77	***NAA25***	42	4	4	19.6
8	***MTHFR***	104	29	10	58	43	***FGF5***	57	3	5	26.8	78	***SUGCT***	28	5	7	19.6
9	***CABCOCO1***	88	58	10	57.3	44	***ST7L***	42	32	5	26.5	79	***TNNT3***	45	6	3	19.4
10	***CSK***	98	19	10	53.9	45	***FURIN***	50	11	5	25.6	80	*UGT2A1*	56	1	1	19.2
11	***NT5C2***	89	45	9	53.5	46	***PDE1A***	39	23	6	25.4	81	***FBN2***	30	5	6	18.6
12	***ARHGAP42***	87	18	12	52.9	47	*PLEKHG1*	46	7	6	25.1	82	***PHACTR1***	36	1	5	18.5
13	***CACNB2***	78	50	10	52	48	***ZFAT***	44	8	6	24.5	83	***XKR6***	22	48	3	18.4
14	***FES***	84	15	11	49.5	49	***AGT***	40	6	7	24.3	84	***ABHD17C***	28	6	6	18.1
15	*SLC39A8*	88	1	10	46.7	50	***ARHGAP24***	35	7	8	24.3	85	*DBH*	34	2	5	17.9
16	***LSP1***	73	24	9	43.6	51	***ESR1***	47	7	5	23.7	86	*PTPN11*	41	6	3	17.9
17	*LMAN1L*	99	9	4	42.2	52	***CFDP1***	32	9	8	23.5	87	***SCAMP2***	36	16	3	17.8
18	*MECOM*	57	36	10	41.4	53	***COL21A1***	36	10	7	23.5	88	***ZNF318***	24	22	5	17.8
19	*CASZ1*	56	10	13	41.4	54	*PINX1*	25	68	3	23.2	89	*ANK2*	52	1	1	17.7
20	***INSR***	66	16	10	41.2	55	***SWAP70***	36	6	7	22.8	90	*VARS*	47	2	2	17.7
21	***GOSR2***	71	2	10	40.5	56	***PLEKHA7***	20	18	9	22.4	91	*FN1*	33	3	5	17.7
22	*RGL3*	82	2	7	39.5	57	***SIPA1***	34	6	7	22	92	***NMT1***	37	4	4	17.7
23	***SOX6***	49	46	9	38.5	58	***GUCY1A1***	32	10	7	22	93	***SBF2***	36	15	3	17.6
24	***MSRA***	49	82	5	38.4	59	*TRAPPC9*	32	9	7	21.8	94	*PFKM*	51	1	1	17.3
25	*NOS3*	77	2	7	37.6	60	*CUX2*	48	3	4	21.7	95	*EBF1*	34	7	4	17.1
26	***NPPA***	81	7	5	36.7	61	*SLC14A2*	43	4	5	21.7	96	***HOXC6***	41	11	2	17.1
27	*CTNNA3*	89	5	3	35.9	62	***AS3MT***	45	18	3	21.6	97	*PDILT*	45	1	2	16.8
28	***KCNK3***	62	11	8	35.3	63	***FGD5***	26	10	8	21.4	98	***CACNA1D***	22	11	6	16.7
29	***CCN3***	86	1	3	34	64	*MYH6*	47	2	4	21.1	99	*VAC14*	44	2	2	16.6
30	***HFE***	57	5	8	32.3	65	*PAX2*	46	4	4	21.1	100	***BORCS7***	35	2	4	16.6
31	*CYP17A1*	71	3	5	32.1	66	***SLC24A3***	36	6	6	21.1	101	*EBF2*	34	4	4	16.5
32	*NOX4*	51	5	9	31.7	67	***COX14***	51	2	3	20.9	102	***JCAD***	30	2	5	16.4
33	***SLC4A7***	23	85	6	30.7	68	***CAPZA1***	41	4	5	20.9	103	***PRDM6***	28	6	5	16.3
34	***NPR3***	75	5	3	30.6	69	*CDK6*	45	3	4	20.5						
35	***SDCCAG8***	41	44	6	30	70	***DOT1L***	30	6	7	20.5						

Genes shown in bold are supported by cis-eQTL measurements (see Additional file 3)

The prioritized gene set is mapped on all human autosomes but chromosomes 13 and 21 (Fig. 3). Notably ~ 45% of the genes are mapped on chromosomes 1 (10 genes), 10 (12 genes), 11 (12 genes) and 12 (11 genes) (Additional file 1: Table S4). Regarding the BP-traits, 47 of the prioritized genes, including the highest scored *ULK4* and *ATP2B1*, are associated with all three of the systolic, diastolic and pulse pressure (Fig. 6, Additional file 2). Finally, 69 of the prioritized genes are also eGenes in at least one of the 27 BP-related tissues (Table 2, Additional file 3), with *ULK4* supported by eQTLs in 25 tissues.

### The BP-associated PPI network reconstruction

#### The GWAS-deduced PPI network

**Fig. 8:**
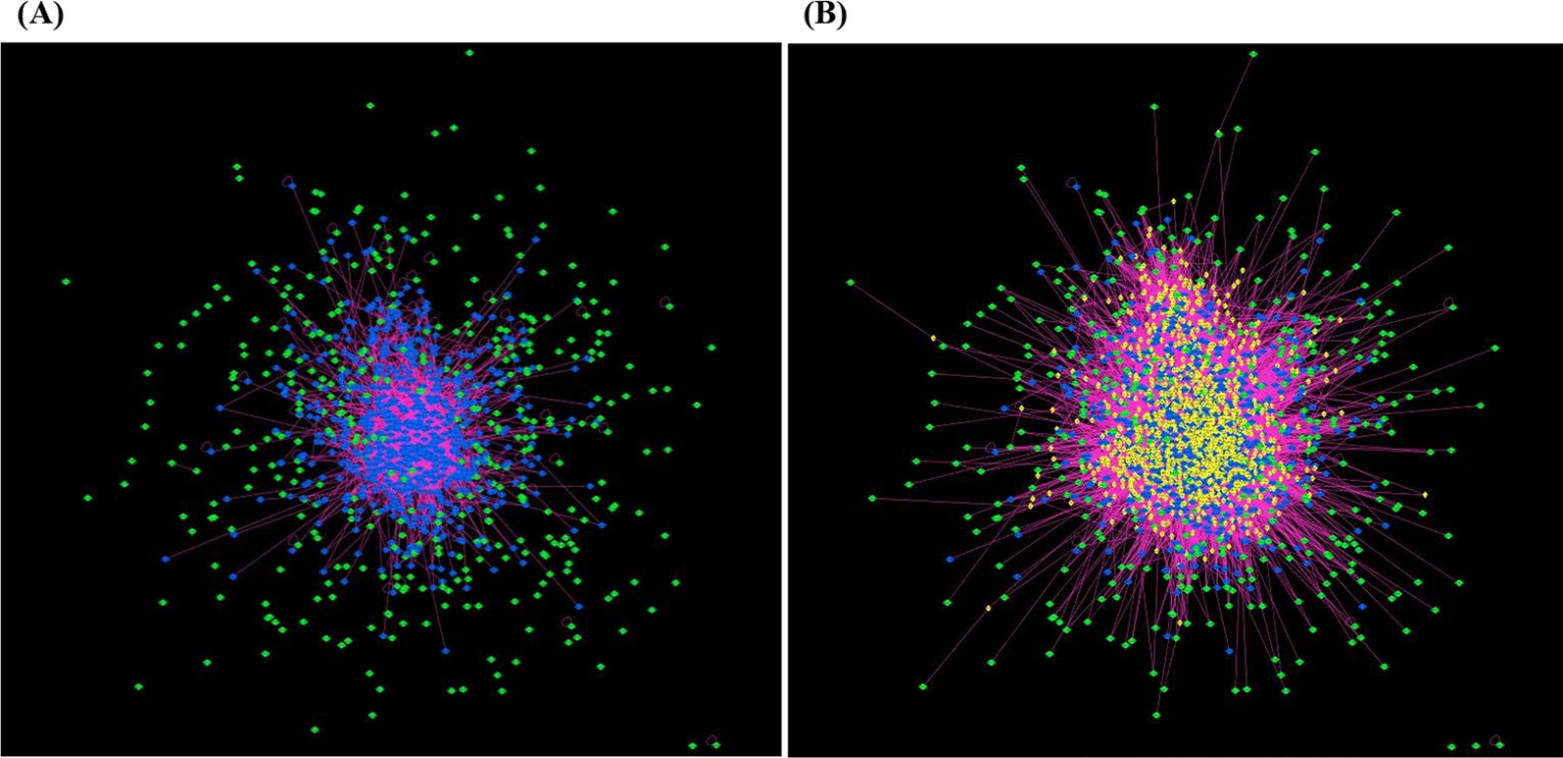
The GWAS-deduced (**A**), and the GWAS-RbSP (**B**) BP PPI networks. Protein-nodes are shown at their position in the force-directed representation of the PICKLE human protein interactome. Nodes are colored based on their type: blue for GWAS-proteins connected in one component (“blue” nodes, BNs); green for the rest of the GWAS-proteins (“green” nodes, GNs) and yellow for the shortest-path intermediates between GNs and BNs (“yellow” nodes, YNs)

In general, the GWAS-deduced PPI network comprises the PPIs between the GWAS-proteins. In our BP-GWAS meta-dataset, 1065 of the 1170 RHCP-proteins have at least one PPI of high-confidence of being direct (Additional file 4). Extracting their PPI subnetwork from the human protein interactome revealed one large component of 672 GWAS-proteins connected through 1700 PPIs (excluding self-interactions) (Additional file 1: Fig. S1A), with most of the rest 393 proteins as 1-mers or homo-dimers and very few in heterodimers (Fig. 8A). The BP-GWAS proteins in the connected component will be referred to as “blue” nodes (BNs) and the rest as “green” nodes (GNs) of the BP-PPI network (Additional file 4). The BN set comprises 372 eGene proteins (55%) and 55 proteins (8%) encoded by GWAS-prioritized genes. The respective numbers in the GN set are 231 (59%) and 34 (~ 9%) (Additional files 3 and 4).

The BN PPI network has a scale-free structure with a very good fit (*R*^2^ =  ~ 91%) (Additional file 1: Figs. S1B, C). P53 is the protein with the highest number of interactions (68), followed by UBC9 (58), ESR1 (56) and FYN (43). The scale-free structure implies that BNs cover a wide range of protein degrees in the human PPI network, in a similar relative representation. Indeed, only seven BNs (1%), i.e., P53, ESR1, UBC9, FYN, TF65, KDM1A and SMAD3, are among the 65 protein-hubs of the human network with > 300 PPIs. The rest of BNs are from all degree zones of the human protein interactome, with ~ 24% having fewer than 11 PPIs (Additional file 4). The particular BN network structure supports the complexity of BP regulation, which is connected to a variety of biological processes.

#### Extending the GWAS-deduced PPI network by the shortest-path approach

Starting from the hypothesis that BNs and GNs should be biologically associated, participating in related molecular processes in the context of BP regulation, we proceeded to investigate their relationship through the identification of the shortest PPI paths that connect GNs to BNs into one connected network. Based on the BN-GN association rationale, there is a high probability for the intermediates of these shortest paths to be BP-associated too (“guilt-by-association”) [18]. The shortest-path intermediates will be referred to as “yellow” nodes, YNs, of the BP PPI network. Hence, we can exploit the human PPI network to enrich the set of the GWAS-identified as BP-associated proteins with the YNs and extend the GWAS-deduced protein interactome with the YN–BN, YN–GN and YN–YN interactions, upgrading and expanding the information that can be derived from GWAS. Thus, the final protein set considered as BP-associated will consist of the GWAS-proteins and the YNs. The extended PPI network, to be referred to as “GWAS-reconstructed by the shortest-path approach” (GWAS-RbSP), will be the corresponding subnetwork of the human protein interactome.

Following our proposed algorithm for the reconstruction of the BP-associated GWAS-RbSP PPI network, we identified 1443 shortest-path intermediates (YNs) (Additional file 4) and observed that almost all GNs are at most second neighbors of a BN, having a common YN neighbor. This result validates our initial hypothesis of a close BN–GN relationship. Finally, the BP-associated GWAS-RbSP PPI network comprises 2505 protein-nodes with 31,439 PPIs (Fig. 8B), providing a considerable extension over the GWAS-deduced network of 672 BNs and 390 GNs with 1700 BN–BN and 7 GN–GN PPIs. The GWAS-RbSP interactome contains 15% and 17%, respectively, of the protein-nodes and PPIs of the human protein interactome, including 62 (7 BNs and 55 YNs) of its 65 hubs (i.e., with > 300 PPIs), further supporting the BP-association with core biological processes (Additional file 1: Fig. S2A). The rest of the proteins cover all degree zones, with 22% (553) having fewer than eleven interactors in the human network (Additional file 4). Network analysis showed that the GWAS-RbSP PPI network follows a scale-free structure with a good fit (*R*^2^ = 83%) (Additional file 1: Figs. S2B, C), even though this had not been necessarily expected due to the specialized way of this network reconstruction. The observed scale-free form may be explained from the fact that the GWAS-RbSP PPI network is reconstructed “around” the scale-free BN network. The amyloid beta A4 protein (ΥΝ; *APP*) is the node with the highest number of interactions (376) in the GWAS-RbSP PPI network, while five more proteins have more than 200 interactors: UBC (YN), P53 (BN, BN network hub), EGFR (YN), ESR1 (BN, GWAS-prioritized, BN network hub) and EP300 (YN) (Additional file 4). All six proteins are hubs (> 300 interactions) of the human protein interactome.

#### Prioritizing BP proteins based on their role in the extended BP PPI network

The role and position of the nodes in the GWAS-RbSP network were evaluated based on their IVI (Eq. 1). Using the cut-off of (Eq. 2), we IVI-prioritized (Criterion 2) 106 proteins (22 BNs, 84YNs) (Table 3, Additional file 4). The most influential nodes of the GWAS-RbSP network were P53 (BN), UBC (YN), ESR1 (BN) and EP300 (YN); all other proteins have IVI lower than 60. Notably, ESR1 is the only common protein with the GWAS-prioritized set; thus, combination of Criteria 1 and 2 extends the BP-prioritized set to 208 proteins in total.

**Table 3:** The prioritized BP-proteins according to their IVI in the GWAS-RbSP BP PPI network (Criterion 2)

Rank	Entry Name	Node Type	IVI	Rank	Entry Name	Node Type	IVI	Rank	Entry Name	Node Type	IVI
1	P53	BN	100.0	37	TRAF2	YN	21.9	73	HS90B	YN	14.5
2	UBC	YN	85.6	38	MK01	BN	20.5	74	1433G	YN	14.4
**3**	**ESR1**	**BN**	**63.9**	39	PML	YN	20.5	75	GSK3B	YN	14.4
4	EP300	YN	60.6	40	HDAC2	YN	20.5	76	TIF1B	YN	14.2
5	A4	YN	57.0	41	SMAD2	YN	20.2	77	KRA59	YN	14.2
6	EGFR	YN	52.9	42	HDAC5	YN	20.2	78	FBW1A	BN	13.8
7	AKT1	YN	47.8	43	ERBB2	YN	19.6	79	IKBA	BN	13.7
8	BRCA1	YN	45.4	44	STAT3	BN	19.4	80	TRI27	YN	13.7
9	CBP	YN	43.9	45	ARF	YN	19.4	81	TAU	BN	13.6
10	HS90A	YN	42.5	46	SQSTM	YN	19.0	82	PPARG	YN	13.6
11	MDM2	YN	41.2	47	HXA1	YN	18.7	83	DISC1	YN	13.2
12	TF65	BN	41.0	48	TLE5	YN	18.7	84	ACTB	YN	13.1
13	ANDR	YN	40.2	49	P85A	YN	18.6	85	LATS2	BN	13.1
14	1433Z	YN	36.7	50	HSP74	BN	18.5	86	SYUA	YN	13.1
15	MYC	YN	34.8	51	KR108	YN	18.2	87	HDAC6	YN	13.0
16	SRC	YN	34.2	52	1433 T	YN	18.0	88	TNR1A	YN	13.0
17	HIF1A	YN	34.1	53	CALM1	YN	18.0	89	NCOR2	BN	12.8
18	CTNB1	YN	34.1	54	CALM2	YN	18.0	90	KDM1A	BN	12.8
19	TRAF6	YN	31.7	55	CALM3	YN	18.0	91	UBE3A	YN	12.7
20	UBC9	BN	30.5	56	RAF1	BN	17.9	92	NEDD4	YN	12.5
21	SP1	YN	29.1	57	IKKB	YN	17.5	93	SIN3A	YN	12.5
22	UBB	YN	27.4	58	H31	YN	16.9	94	PTEN	BN	12.3
23	HSP7C	YN	27.0	59	KAT2B	YN	16.8	95	LNX1	YN	12.1
24	ABL1	YN	26.0	60	H31T	YN	16.6	96	XRCC6	BN	12.0
25	PARP1	YN	25.7	61	HDAC4	BN	16.5	97	LRRK2	YN	12.0
26	RL40	YN	25.0	62	NT2NA	YN	16.2	98	M3K3	YN	12.0
27	GRB2	YN	23.4	63	NPM	YN	16.1	99	UB2D1	YN	11.9
28	CHIP	YN	23.1	64	FYN	BN	15.9	100	DDB1	YN	11.8
29	GCR	YN	23.0	65	CUL1	YN	15.8	101	SCNM1	YN	11.7
30	JUN	YN	22.8	66	H4	BN	15.6	102	UB2D2	YN	11.6
31	SMAD3	BN	22.6	67	CDN1A	BN	15.5	103	SUMO1	YN	11.6
32	CSK21	YN	22.3	68	CDK1	YN	15.4	104	TERA	YN	11.5
33	NEMO	YN	22.3	69	RB	YN	15.3	105	PCNA	YN	11.5
34	RS27A	YN	22.3	70	1433E	YN	15.2	106	ATL4	YN	11.5
35	SMCA4	YN	22.0	71	KAT5	BN	14.7				
36	CRTP1	YN	21.9	72	H2AX	YN	14.7				

The Protein Entry Name by UniProt is shown without the _HUMAN extension; BN: “Βlue Node” denotes a BP GWAS-protein among those connected in one large PPI component; GN: “Green Node” denotes any other BP GWAS-protein of the human protein interactome, and YN: “Yellow Node” denotes a shortest-path intermediate between GNs and BNs

ESR1 is shown in bold as the only protein in this set that was also GWAS-prioritized (Table 2)

By the IVI definition (Eq. 1), the set of the IVI-prioritized proteins of the BP PPI network was indeed expected to be mainly populated with the top-scored in the “hubness” feature of the IVI (Additional file 4). However, it is of value to mention the protein-nodes with the highest scores in the first and second part of the “spreading” index (Eq. 1), as they may reveal specialized players in BP regulation, which are not directly apparent when only the comprehensive IVI of a protein-node is considered (Additional file 4). The four top-scored in the first “spreading” part, which reveals semi-local hubs too, are: SP1 (YN), MINY4 (BN), AKT1 (YN) and HIF1A (YN); all but MINY4 are in the IVI-prioritized set. The top-scored in the second part of the “spreading” index, which reveals proteins important in maintaining the connectivity of the network, are: A4 (YN), AQP6 (YN, not IVI-prioritized), F209A (YN, not IVI-prioritized) and GP152 (YN, not IVI-prioritized).

#### Prioritizing YNs through their association with GWAS-prioritized proteins

The novel, second network-based prioritization criterion that we proposed promotes the YNs that are common neighbors of GWAS-prioritized proteins. More specifically, we identified the YNs that are common interactors of any two of the 88 (55 BNs, 33 GNs) GWAS-prioritized proteins included in the GWAS-RbSP BP PPI network, and then, we isolated the subnetwork of these YNs and their GWAS-prioritized neighbors (Additional file 1: Fig. S3). The protein-nodes of this final subnetwork were considered prioritized according to Criterion 3. They included 175 YNs and 78 (50 BNs, 28 GNs) GWAS-prioritized proteins (Additional file 5); 48 of the YNs were also IVI-prioritized (Criterion 2). The IVI-ranking of the 253 proteins in this interactome is shown in Table 4. ESR1 (BN) is the protein with the highest IVI, followed by three YNs (AKT1, EGFR and CTNB1) with an IVI higher than 50. In total, only 15 proteins (ESR1 and 14 YNs) had an IVI higher than the significance cutoff of Eq. 2 (i.e., > 25), all of which were also IVI-prioritized in the GWAS-RbSP PPI network. The next two highest ranked BNs are INSR and PTN11, in the 24th and 25th positions, respectively.

**Table 4:** The IVI-ranked proteins in the network of the GWAS-prioritized and their common YNs (Criterion 3)

Rank	Entry Name	Node Type	IVI	Rank	Entry Name	Node Type	IVI	Rank	Entry Name	Node Type	IVI	Rank	Entry Name	Node Type	IVI	Rank	Entry Name	Node Type	IVI	Rank	Entry Name	Node Type	IVI
**1**	ESR1*	**BN**	**100.0**	44	SUMO2	YN	14.6	87	RACK1	YN	7.7	130	RASH	YN	5.2	173	SRSF2	YN	2.4	216	PHAR1	GN	1.3
**2**	**AKT1**	**YN**	**77.0**	**45**	**HDAC2**	**YN**	**13.8**	88	PTPRJ	YN	7.6	131	SH3G2	YN	5.1	174	CCN2	YN	2.3	217	PLCE1	GN	1.3
**3**	**EGFR**	**YN**	**63.0**	46	KIT	YN	13.1	89	E2F1	YN	7.5	132	APC	YN	5.0	175	MAPK2	YN	2.3	218	FGF5	GN	1.3
**4**	**CTNB1**	**YN**	**51.0**	47	MK03	YN	12.9	90	CHK2	YN	7.5	133	CADH2	YN	5.0	176	SKIL	YN	2.3	219	CO8A1	YN	1.2
**5**	**UBC**	**YN**	**46.3**	48	STK11	YN	12.8	91	DHX9	YN	7.5	**134**	**LNX1**	**YN**	**5.0**	177	HXC6	BN	2.2	220	CN119	YN	1.2
**6**	**BRCA1**	**YN**	**42.2**	49	ERBB3	YN	12.6	92	RPB1	YN	7.4	135	SH2B3	BN	4.7	178	ATX2	BN	2.2	221	CUX2	GN	1.2
**7**	**GRB2**	**YN**	**39.0**	50	XRCC5	YN	12.6	93	FOS	YN	7.3	136	PTPA	YN	4.6	179	PINX1	BN	2.2	222	SOX6	BN	1.2
**8**	**A4**	**YN**	**37.3**	51	LCK	YN	12.5	94	KPCB	YN	7.3	137	1433F	YN	4.6	180	ADRB1	GN	2.2	223	CAD13	BN	1.2
**9**	**SRC**	**YN**	**35.3**	52	IGF1R	YN	12.4	95	CSN5	YN	7.2	138	AT2B1	BN	4.3	181	AAPK2	YN	2.2	224	BBS2	YN	1.2
**10**	**EP300**	**YN**	**33.7**	**53**	**TERA**	**YN**	**12.3**	96	RARA	YN	7.1	139	H2B1H	YN	4.1	182	SWP70	GN	2.1	225	NAA25	GN	1.2
**11**	**CBP**	**YN**	**33.1**	**54**	**H31**	**YN**	**11.9**	97	SMUF1	YN	7.0	140	FES	BN	4.1	183	PKHA7	BN	2.1	226	CD209	YN	1.2
**12**	**ERBB2**	**YN**	**31.5**	55	PK3CA	YN	11.8	98	HD	YN	7.0	141	RAD51	YN	4.1	184	MYOME	YN	2.1	227	TPPC9	GN	1.2
**13**	**ANDR**	**YN**	**29.5**	56	CDK6	BN	11.8	99	ARRB1	YN	6.9	142	FINC	BN	4.1	185	MAGI1	YN	2.0	228	TPC2A	YN	1.2
**14**	**HIF1A**	**YN**	**28.5**	**57**	**RB**	**YN**	**11.5**	100	PAXI	YN	6.9	143	PAXI1	YN	4.0	186	PAX2	BN	1.9	229	ARVC	GN	1.2
**15**	**1433Z**	**YN**	**27.6**	58	CRKL	YN	11.4	101	FGFR2	YN	6.8	144	PRDM6	BN	3.5	187	ECHP	YN	1.9	230	FBN2	BN	1.1
**16**	**SP1**	**YN**	**26.2**	59	ITA4	YN	11.4	102	H11	YN	6.7	145	ENPL	YN	3.4	188	STX1A	YN	1.8	231	TULP3	YN	1.1
**17**	**CSK21**	**YN**	**24.9**	60	KHDR1	YN	11.4	103	CACO2	YN	6.6	146	SIAH1	YN	3.3	189	TRI63	YN	1.8	232	HACD3	YN	1.1
**18**	**HS90A**	**YN**	**24.7**	61	CSK	BN	11.0	104	PP2AA	YN	6.6	147	CP17A	GN	3.3	190	GCYA1	BN	1.8	233	CACB2	BN	1.1
**19**	**TRAF6**	**YN**	**24.5**	62	FGFR4	YN	10.9	105	HNRPU	YN	6.6	148	LRRK1	YN	3.2	191	CTNA3	BN	1.8	234	CCN3	BN	1.1
**20**	**P85A**	**YN**	**23.2**	63	FZR1	YN	10.8	106	NOS3	BN	6.5	149	ERBIN	YN	3.1	192	NMT1	BN	1.8	235	ERO1A	YN	1.1
**21**	**PARP1**	**YN**	**22.8**	**64**	**H31T**	**YN**	**10.7**	107	TSC1	YN	6.5	150	CBY1	YN	3.1	193	ARF1	YN	1.8	236	FURIN	BN	1.1
**22**	**CHIP**	**YN**	**22.5**	65	PP1G	YN	10.5	108	STA5A	YN	6.4	151	PDE1A	BN	3.0	194	5NTC	BN	1.7	237	ZFAT	GN	1.1
**23**	**MYC**	**YN**	**22.0**	66	CDC37	YN	10.4	109	ANM1	YN	6.3	152	VAC14	BN	3.0	195	BKRB2	YN	1.7	238	CAC1D	GN	1.0
24	INSR	BN	21.9	67	SH3K1	YN	10.4	110	HNRPC	YN	6.3	153	LASP1	YN	2.9	196	ULK4	GN	1.7	239	ANPRC	BN	1.0
25	PTN11	BN	21.5	**68**	**HS90B**	**YN**	**10.4**	111	HNF4A	YN	6.3	154	ARI5A	YN	2.9	197	MYH6	BN	1.6	240	CASZ1	BN	1.0
**26**	**SMAD2**	**YN**	**21.5**	69	AURKA	YN	10.0	112	CADH1	YN	6.1	155	DOT1L	BN	2.9	198	RASN	YN	1.6	241	ANGT	BN	1.0
**27**	**NEMO**	**YN**	**21.2**	**70**	**1433E**	**YN**	**9.9**	113	ACTN2	YN	6.1	156	CAZA1	BN	2.8	199	SDCG8	BN	1.6	242	FTO	BN	1.0
**28**	**NPM**	**YN**	**20.9**	71	EPHA2	YN	9.7	114	FKBP4	YN	6.0	157	FBX7	YN	2.8	200	MTMRD	BN	1.5	243	SCAM2	BN	1.0
**29**	**UBB**	**YN**	**19.6**	72	HGS	YN	9.5	115	TS101	YN	6.0	158	PFKAM	BN	2.7	201	CFDP1	BN	1.5	244	WNT2B	GN	1.0
**30**	**ARF**	**YN**	**19.6**	**73**	**TIF1B**	**YN**	**9.5**	116	ADRB2	YN	5.7	159	MTR1A	YN	2.7	202	GOSR2	BN	1.5	245	CLCN6	GN	1.0
**31**	**RL40**	**YN**	**19.4**	**74**	**PCNA**	**YN**	**9.2**	117	LMNA	YN	5.7	160	HFE	GN	2.7	203	KLK6	YN	1.5	246	AB17C	GN	1.0
**32**	**HSP7C**	**YN**	**19.2**	75	FGFR1	YN	9.0	118	TERF1	YN	5.7	161	CC120	YN	2.7	204	LSP1	GN	1.5	247	COX14	GN	1.0
**33**	**RS27A**	**YN**	**19.0**	76	IKKE	YN	9.0	119	EZH2	YN	5.6	162	PO6F2	YN	2.7	205	COE1	BN	1.5	248	COE2	BN	1.0
**34**	**CALM1**	**YN**	**18.6**	77	PP1A	YN	8.9	120	TNIK	YN	5.6	163	SH3G3	YN	2.7	206	MBD5	GN	1.4	249	LMA1L	GN	1.0
**35**	**CALM2**	**YN**	**18.6**	78	ARRB2	YN	8.9	121	KAPCA	YN	5.6	164	SOX10	YN	2.6	207	RHG24	GN	1.4	250	UT2	GN	1.0
**36**	**CALM3**	**YN**	**18.6**	**79**	**DISC1**	**YN**	**8.8**	122	DDT4L	YN	5.5	165	PPIB	YN	2.6	208	PDGFA	YN	1.4	251	MSRA	BN	1.0
37	PLCG1	YN	17.8	80	HSPB1	YN	8.8	123	1433B	YN	5.5	166	KSR1	YN	2.5	209	ANF	BN	1.4	252	RGL3	GN	1.0
**38**	**JUN**	**YN**	**17.7**	81	FHL2	YN	8.7	124	NOS1	YN	5.4	167	KCNK3	GN	2.5	210	CE164	BN	1.4	253	PDILT	GN	1.0
**39**	**ACTB**	**YN**	**16.9**	82	FLNA	YN	8.7	125	H33	YN	5.4	168	SYVC	BN	2.5	211	ZN318	GN	1.4				
40	PGFRA	YN	15.6	83	LYN	YN	8.6	126	ANM5	YN	5.4	169	CCHCR	YN	2.5	212	EXOS5	YN	1.3				
**41**	**1433T**	**YN**	**15.3**	84	NUCL	YN	8.5	127	CDK7	YN	5.4	170	MECOM	BN	2.4	213	ANK2	BN	1.3				
42	PTN6	YN	14.7	85	IRS1	YN	8.1	128	CBX8	YN	5.2	171	HXC4	BN	2.4	214	TNNT3	GN	1.3				
**43**	**TNR1A**	**YN**	**14.7**	86	SNAI1	YN	8.0	129	H12	YN	5.2	172	THOC4	YN	2.4	215	RL7A	YN	1.3				

BN, GN, YN are used as explained in the notes of Table 3; The Protein Entry Name by UniProt is shown without the _HUMAN extension

Proteins shown in bold are also IVI-prioritized in the GWAS RbSP PPI Network; (*) ESR1 is the only protein prioritized by all three criteria (Tables 2 and 3)

### Ranking the complete set of prioritized BP-associated proteins

Overall, 335 BP-proteins were prioritized based on any of the three prioritization criteria (Fig. 1, Additional file 1: Fig. S4, Additional file 5), i.e., 103 proteins according to the GWAS-based Criterion 1, 106 proteins according to the network-based Criterion 2 and 253 proteins according to the network-based Criterion 3. We proposed to rank the prioritized protein-set based on the number of satisfied prioritization criteria. ESR1 (BN) was ranked at the top as the only protein prioritized based on all three criteria, underlining its high ranking in the network-based Criterion 2 (3rd) and Criterion 3 (1st) (Tables 2, 3 and 4). Additional 77 (49 BNs, 28 GNs) GWAS-prioritized proteins (Criterion 1) had common YN interactors (Criterion 3) and 48 YNs were prioritized by both network-based Criteria 2 and 3 (Table 4, Additional file 5). The rest 209 BP-proteins were prioritized based on a single criterion. In the protein group with two satisfied prioritization criteria, we proposed to rank higher the 77 GWAS-prioritized proteins with common YN interactors compared to the 48 YNs prioritized by both network-based criteria. In both subgroups, the internal ranking was made according to the network-based Criterion 3. Finally, in the single criterion group, the 26 GWAS-prioritized proteins (Criterion 1) were ranked higher than the 127 YNs of Criterion 3, leaving last the 57 BP-proteins (BNs and YNs) of Criterion 2. Based on this ranking scheme, the nine proteins following ESR1 in the BP-associated top-10 are all GWAS-prioritized BNs: INSR, PTN11, CDK6, CSK, NOS3, SH2B3, ATP2B1, FES and FINC (Table 5, Additional file 5). Interestingly, INSR, PTN11, CDK6, NOS3, FES and FINC emerged among the most BP-significant due to the network-based Criterion 3, while in the GWAS-prioritized list ranked from position 14 (for FES), to position 91 (for FINC). INSR and FES are also supported by eQTLs in 5 and 10 BP-related tissues, respectively.

**Table 5:** The top-10 BP-prioritized proteins

Protein Entry Name	Gene Symbol	Criterion 1	Criterion 2	Criterion 3	Overall Ranking
ESR1^BN^	*ESR1*	✓	✓	✓	1
INSR^BN^	*INSR*	✓		✓	2
PTN11^BN^	*PTPN11*	✓		✓	3
CDK6^BN^	*CDK6*	✓		✓	4
CSK^BN^	*CSK*	✓		✓	5
NOS3^BN^	*NOS3*	✓		✓	6
SH2B3^BN^	*SH2B3*	✓		✓	7
ATP2B1^BN^	*ATP2B1*	✓		✓	8
FES^BN^	*FES*	✓		✓	9
FINC^BN^	*FN1*	✓		✓	10

The Protein Entry Name by UniProt is shown without the _HUMAN extension

^BN^denotes a Blue Node (BN) as described in the Notes of Table 3

✓ denotes a satisfied criterion

**Fig. 9:**
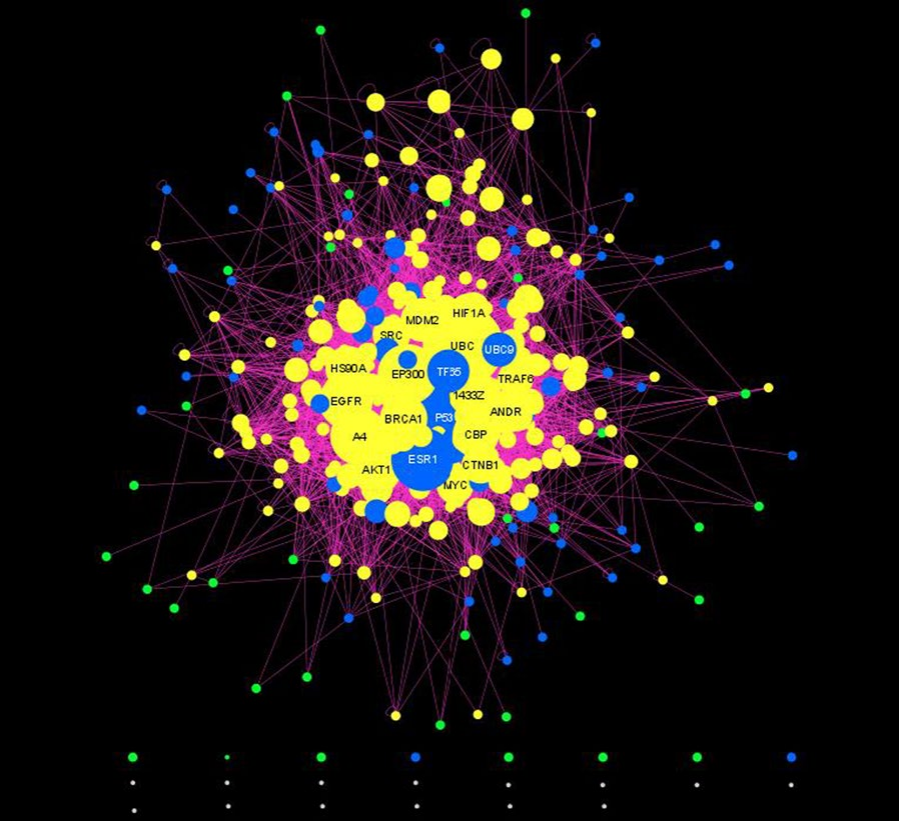
The prioritized BP-protein PPI network. Nodes are color-coded as explained in Fig. 8. Node size corresponds to the IVI of the protein in the GWAS-RbSP PPI network. The UniProt Entry Names (excluding the _HUMAN extension) of the protein nodes with the highest IVI are shown

We observed that 93% (313; 74 BNs, 28 GNs, 243 YNs) of the 335 prioritized BP-proteins are connected through a network of 3868 PPIs (excluding self-loops), as shown in Fig. 9. From the 126 BP-proteins prioritized by at least two criteria, 111 (88%, 46 BNs, 17 GNs, 48 YNs) form a connected network of 642 interactions (excluding self-loops). The genes of the 335 prioritized BP-associated proteins map on all chromosomes but Y, revealing chromosomes 1, 12, 17 and 11 as the most enriched, with 33, 25, 25 and 24 prioritized genes, respectively. In the group of the 126 BP-proteins backed by two prioritization criteria, all chromosomes but Y are represented, with most enriched chromosomes 12, 1 and 11 with 13, 11, and 9 genes, respectively. These observations further support the higher enrichment of chromosomes 1, 11 and 12 in BP-associated genes, which was indicated from the GWAS-genes too.

### Pathway enrichment analysis

Pathway and functional analyses were performed on the full set of the 2613 BP-associated proteins. KEGG pathway enrichment analysis indicated that about half of the BP-proteins belong to at least one of 314 KEGG-defined pathways. Eighty-seven (28%) of these pathways are significantly enriched in BP-proteins according to DAVID (i.e., *q* < 0.05) (Table 6). The BP-enriched pathways include the dilated (DCM), hypertrophic (HCM) and arrhythmogenic right ventricular cardiomyopathy (ARVC) pathways, thirty (30) signaling pathways, among which the adrenergic signaling in cardiomyocytes and the PI3K-Akt, the Rap1, the cGMP-PKG, the cAMP, the HIF-1 (hypoxia-inducible factor 1) and the calcium signaling pathways, four (4) focal adhesion/axon guidance-related pathways, the vascular smooth muscle contraction and the regulation of actin cytoskeleton pathway. The BP-associated ‘aldosterone synthesis and secretion’, ‘renin secretion’, ‘insulin resistance’, ‘insulin secretion’ and ‘thyroid hormone synthesis’ pathways were also identified among the significantly BP-enriched. Notably, the 87-pathway list includes thirty (30) cancer or viral/bacterial infection-associated pathways.

**Table 6:** The BP-enriched KEGG pathways

	KEGG ID/Pathway	# Proteins	FDR		KEGG ID/Pathway	# Proteins	FDR		KEGG ID/Pathway	# Proteins	FDR
1	hsa05200 Pathways in cancer	189	1.1E−30	**31**	**hsa05218 Melanoma**	**36**	**1.4E−06**	61	hsa04360 Axon guidance	66	3.2E−04
2	hsa05215 Prostate cancer	61	6.3E−20	**32**	**hsa04110 Cell cycle**	**50**	**1.4E−06**	**62**	**hsa05230 Central carbon metabolism in cancer**	**29**	**4.6E−04**
3	hsa04151 PI3K-Akt signaling pathway	136	8.6E−18	33	hsa04390 Hippo signaling pathway	57	1.5E−06	**63**	**hsa05168 Herpes simplex virus 1 infection**	**86**	**5.1E−04**
**4**	**hsa05203 Viral carcinogenesis**	**86**	**1.8E−14**	34	hsa04066 HIF-1 signaling pathway	43	2.1E−06	**64**	**hsa05160 Hepatitis C**	**60**	**5.2E−04**
5	hsa04520 Adherens junction	45	1.4E−13	**35**	**hsa05211 Renal cell carcinoma**	**34**	**2.9E−06**	65	hsa04310 Wnt signaling pathway	52	1.7E−03
6	hsa04915 Estrogen signaling pathway	66	2.9E−13	36	hsa04921 Oxytocin signaling pathway	61	3.5E−06	66	hsa04370 VEGF signaling pathway	27	2.2E−03
7	hsa04540 Gap junction	51	7.3E−13	37	hsa04071 Sphingolipid signaling pathway	48	4.3E−06	**67**	**hsa04668 TNF signaling pathway**	**39**	**2.6E−03**
8	hsa05166 Human T cell leukemia virus 1 infection	109	1.0E−12	**38**	**hsa04014 Ras signaling pathway**	**77**	**6.3E−06**	**68**	**hsa05219 Bladder cancer**	**21**	**2.7E−03**
9	hsa04068 FoxO signaling pathway	62	5.7E−12	**39**	**hsa04810 Regulation of actin cytoskeleton**	**72**	**7.6E−06**	**69**	**hsa04660 T cell receptor signaling pathway**	**39**	**3.3E−03**
10	hsa04015 Rap1 signaling pathway	89	6.2E−12	40	hsa05213 Endometrial cancer	30	7.9E−06	**70**	**hsa04920 Adipocytokine signaling pathway**	**29**	**4.1E−03**
**11**	**hsa04010 MAPK signaling pathway**	**109**	**7.2E−12**	41	hsa04261 Adrenergic signaling in cardiomyocytes	58	9.8E−06	71	hsa05100 Bacterial invasion of epithelial cells	31	4.8E−03
12	hsa05161 Hepatitis B	71	7.5E−12	42	hsa05210 Colorectal cancer	41	9.9E−06	72	hsa04062 Chemokine signaling pathway	59	5.0E−03
13	hsa04510 Focal adhesion	84	1.9E−11	**43**	**hsa05223 Non**−**small cell lung cancer**	**35**	**1.2E−05**	73	hsa04914 Progesterone−mediated oocyte maturation	34	7.0E−03
14	hsa04022 cGMP-PKG signaling pathway	72	1.9E−11	44	hsa05231 Choline metabolism in cancer	42	1.3E−05	74	hsa04666 Fc gamma R-mediated phagocytosis	33	9.0E−03
15	hsa05220 Chronic myeloid leukemia	43	8.6E−11	45	hsa05202 Transcriptional misregulation in cancer	61	1.3E−05	75	hsa04911 Insulin secretion	34	1.2E−02
16	hsa05205 Proteoglycans in cancer	80	1.1E−10	**46**	**hsa04550 Signaling pathways regulating pluripotency of stem cells**	**53**	**1.7E−05**	**76**	**hsa04720 Long-term potentiation**	**29**	**1.3E−02**
**17**	**hsa04919 Thyroid hormone signaling pathway**	**56**	**2.0E−10**	47	hsa04730 Long-term depression	32	1.8E−05	77	hsa04918 Thyroid hormone synthesis	30	1.4E−02
18	hsa04725 Cholinergic synapse	51	3.0E−09	48	hsa05414 Dilated cardiomyopathy (DCM)	43	1.9E−05	**78**	**hsa05145 Toxoplasmosis**	**39**	**1.5E−02**
19	hsa04024 cAMP signaling pathway	82	5.9E−09	49	hsa04925 Aldosterone synthesis and secretion	47	2.3E−05	**79**	**hsa04380 Osteoclast differentiation**	**43**	**1.5E−02**
**20**	**hsa05214 Glioma**	**39**	**1.1E−08**	50	hsa04924 Renin secretion	37	2.5E−05	80	hsa04020 Calcium signaling pathway	60	1.5E−02
**21**	**hsa04120 Ubiquitin mediated proteolysis**	**60**	**6.3E−08**	51	hsa05410 Hypertrophic cardiomyopathy (HCM)	40	2.9E−05	81	hsa05412 Arrhythmogenic right ventricular cardiomyopathy (ARVC)	30	1.8E−02
22	hsa05221 Acute myeloid leukemia	33	6.7E−08	52	hsa04916 Melanogenesis	45	3.2E−05	82	hsa04917 Prolactin signaling pathway	29	1.9E−02
23	hsa05222 Small cell lung cancer	43	9.1E−08	53	hsa04931 Insulin resistance	45	3.8E−05	83	hsa04922 Glucagon signaling pathway	40	2.0E−02
24	hsa04728 Dopaminergic synapse	59	1.3E−07	**54**	**hsa04350 TGF-beta signaling pathway**	**38**	**7.2E−05**	84	hsa04664 Fc epsilon RI signaling pathway	28	2.4E−02
25	hsa04012 ErbB signaling pathway	41	2.2E−07	**55**	**hsa04114 Oocyte meiosis**	**46**	**9.6E−05**	85	hsa04270 Vascular smooth muscle contraction	48	2.8E−02
**26**	**hsa05212 Pancreatic cancer**	**37**	**3.5E−07**	56	hsa05142 Chagas disease (American trypanosomiasis)	43	1.2E−04	**86**	**hsa05130 Pathogenic *Escherichia coli* infection**	**64**	**4.1E−02**
**27**	**hsa05169 Epstein–Barr virus infection**	**68**	**7.2E−07**	**57**	**hsa04662 B cell receptor signaling pathway**	**32**	**2.1E−04**	87	hsa04750 Inflammatory mediator regulation of TRP channels	38	4.3E−02
**28**	**hsa05216 Thyroid cancer**	**23**	**7.9E−07**	58	hsa04713 Circadian entrainment	42	2.4E−04				
29	hsa04912 GnRH signaling pathway	46	1.2E−06	**59**	**hsa04144 Endocytosis**	**75**	**2.9E−04**		hsa01100 Metabolic pathways	140	1.00 + 02
**30**	**hsa04722 Neurotrophin signaling pathway**	**51**	**1.3E−06**	60	hsa04710 Circadian rhythm	19	3.0E−04				

The names of the pathways with >65% of their proteins being YNs are shown in bold

The pathway name is preceded by the pathway KEGG ID; hsa denotes *Homo sapiens*

The false discovery rate (FDR) was calculated by DAVID

**Fig. 10:**
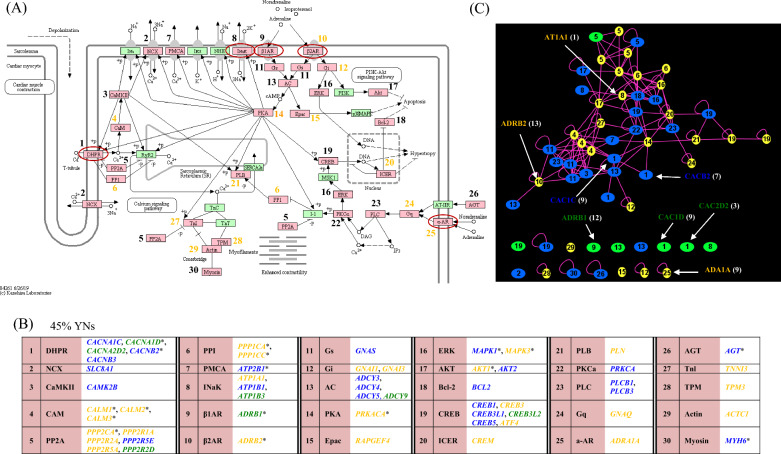
The BP-proteins in the KEGG-defined adrenergic signaling in cardiomyocytes pathway and their PPI network. The BP-proteins are shown (**A**) in the context of the KEGG pathway map, (**B**) with their gene symbols in respect to the numbered pathway map node symbols, and (**C**) in their subnetwork in the GWAS-RbSP protein interactome. BP-proteins in (**C**) are denoted by the number of the associated pathway map node. The color code in (**A**–**C**) indicates the protein-type (BN, GN, YN) as explained in Fig. 8. The prioritized BP-proteins are denoted with an asterisk in (**B**). Τhe proteins that are antihypertensive drug targets are indicated with a red eclipse in (**A**) and by their UniProt entry name in (**C**); the number of the targeting drugs is shown in parenthesis (Additional file 4)

**Fig. 11:**
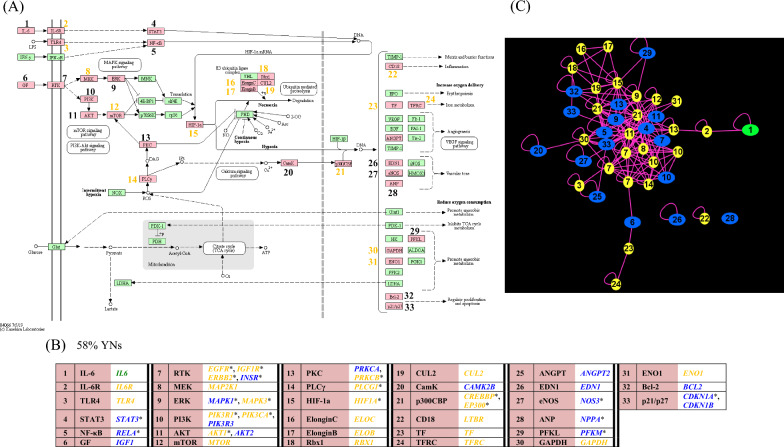
The BP-proteins in the KEGG-defined HIF-1 signaling pathway and their PPI network. The BP proteins are shown (**A**) in the context of the KEGG pathway map, (**B**) with their gene symbols in respect to the numbered pathway map node symbols, and (**C**) in their subnetwork of the GWAS-RbSP protein interactome. BP-proteins in (**C**) are denoted by the number of the associated pathway map node. The figure is color-coded and structured as described in the legend of Fig. 10

To investigate and validate the significance of the PPI network-deduced YN proteins in BP regulation and connect the protein interactome to BP functional information, we selected four of the significantly BP-enriched KEGG-defined pathways, i.e., adrenergic signaling in cardiomyocytes (Fig. 10), HIF-1 signaling (Fig. 11), cGMP-PKG signaling (Additional file 1: Fig. S5) and DCM (Additional file 1: Fig. S6), which have been directly associated with hypertension and/or heart pathophysiology, e.g., [53–59]. The selected pathways integrate also parts of the PI3K-Akt, calcium and cAMP signaling, the vascular smooth muscle contraction, insulin resistance, insulin secretion and renin secretion pathways. To investigate any BP-associated metabolic mechanisms, we also considered the KEGG-defined “Metabolic Pathways” (Additional file 1: Fig. S7). In general, in 31 (~ 36%) of the 87 BP-enriched KEGG pathways, YNs constitute more than 65% of the involved proteins. In all cases, YNs enhance the statistical significance of the BP-association of the pathways, while pathways such as the HIF-1 signaling would not have been revealed as BP-enriched if only the GWAS-proteins had been considered. Moreover, there are numerous YNs, which are proteins of crucial role in BP-associated functional pathways, validating thus their BP-association and supporting the pursued network-based analysis of the GWAS data that revealed this association. Some characteristic ΥΝ examples are: HIF1A (central protein of the HIF-1 signaling pathway (Fig. 11)), ADRB2, GNAI1, GNAI3, GNAQ, ADΑ1A (proteins involved in the adrenergic signaling in cardiomyocytes pathway (Fig. 10)), KAPCA, PPLA, TNNI3, TPM3, ACTC (proteins involved both in the adrenergic signaling in cardiomyocytes (Fig. 10) and the DCM (Additional file 1: Fig. S6) pathways), and LMNA and ACTB (proteins involved in the DCM pathway (Additional file 1: Fig. S6)). Furthermore, 39% of the proteins in the “Metabolic Pathways” are YNs, contributing to the elucidation of the steroid hormone synthesis, the biosynthesis of unsaturated fatty acids, the fatty acid elongation in mitochondria and the purine metabolism as BP-associated based on the GWAS data (Additional file 1: Fig. S7B). Not significant conclusions could have been made about these pathways if only the GWAS proteins had been mapped on the KEGG Metabolic Pathways (Additional file 1: Fig. S7A).

Pathway enrichment analysis of the 335 BP-prioritized protein group indicated similar enrichment as in the overall BP-protein set for the PI3K-AKT signaling, MAPK signaling, Ras signaling, focal adhesion, estrogen signaling and regulation of actin cytoskeleton pathways. On the other hand, the BP-prioritized set showed higher enrichment over the full BP-protein set in the thyroid hormone and neurotrophin signaling pathways.

### BP-proteins as antihypertensive drug targets

A total of 61 antihypertensive drugs were identified in DrugBank, targeting 34 BP proteins (13 BNs, 10 GNs, 11 YNs) (Additional file 4), 8 of which belong to the prioritized set (Additional file 5): PDE1A (BN), ADRB1 (GN), CACB2 (BN), CAC1D (GN), JUN (YN), MTHR (GN), ADRB2 (YN), PPARG (YN). For the 11 YNs, which are antihypertensive drug targets, this is an additional validation of their association with BP. Twenty-five out of the 34 proteins are involved in 47 BP-enriched KEGG pathways and seven (four uniquely) in “Metabolic Pathways”. Three antihypertensive drug targets, two in the prioritized set, are involved in at least 17 BP-enriched pathways: JUN (24), CAC1C (BN; 18) and CAC1D (17). On the other hand, the calcium and cGMP-PKG signaling pathways are the most enriched with antihypertensive drug BP-protein targets (i.e., 10 targets). ‘Renin secretion’, ‘cAMP signaling’, ‘Adrenergic signaling in cardiomyocytes’, ‘Vascular smooth muscle contraction’, ‘Insulin secretion’ and ‘Oxytocin signaling pathway’ complete the set of BP-enriched KEGG pathways involving at least 6 antihypertensive drug targets (Table 7).

**Table 7:** The BP-enriched KEGG pathways involving protein targets of at least nine antihypertensive drugs

BP-enriched KEGG pathway	Number of Antihypertensive drugs targeting pathway proteins	Number of Antihypertensive BP-protein targets
hsa04924 Renin secretion	45	9
hsa04022 cGMP-PKG signaling pathway	39	10
hsa04020 Calcium signaling pathway	32	10
hsa04024 cAMP signaling pathway	30	9
hsa04261 Adrenergic signaling in cardiomyocytes	29	8
hsa04270 Vascular smooth muscle contraction	25	6
hsa05410 Hypertrophic cardiomyopathy (HCM)	24	5
hsa01100 Metabolic pathways	23	7
hsa05414 Dilated cardiomyopathy (DCM)	21	5
hsa05142 Chagas disease (American trypanosomiasis)	16	2
hsa04911 Insulin secretion	13	6
hsa04540 Gap junction	12	1
hsa04921 Oxytocin signaling pathway	12	6
hsa04925 Aldosterone synthesis and secretion	12	4
hsa04010 MAPK signaling pathway	10	5
hsa04725 Cholinergic synapse	10	5
hsa04728 Dopaminergic synapse	10	3
hsa04912 GnRH signaling pathway	10	3
hsa04713 Circadian entrainment	9	2
hsa04720 Long-term potentiation	9	1
hsa05412 Arrhythmogenic right ventricular cardiomyopathy (ARVC)	9	4

**Fig. 12:**
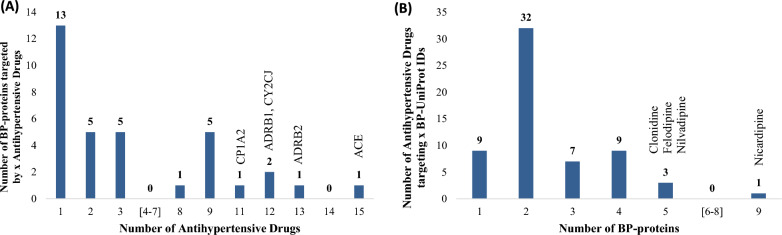
Distribution of the antihypertensive drugs regarding their BP-protein targets (**A**) and of the BP-proteins regarding targeting drugs (**B**)

Eleven BP-proteins are targeted by at least 8 drugs each, fifteen being the maximum number of drugs targeting one protein, ACE (BN) (Fig. 12A). The rest of the proteins (23) are targeted by at most 3 drugs and thirteen of them are targets of only one drug. At the pathway level, 20 BP-enriched KEGG pathways and the “Metabolic Pathways” are targeted by at least 9 antihypertensive drugs (Table 7). Four pathways involve protein targets of at least 30 drugs: Renin secretion (45), cGMP-PKG signaling (39), calcium signaling (32) and cAMP signaling (30). The vast majority (41) of the 61 antihypertensive drugs target at most two BP-proteins (Fig. 12B). Nicardipine, a calcium channel blocker (DrugBank ID:DB00622), has the maximum number of BP-protein targets (9), followed by clonidine, “an agonist of alpha-2 adrenoceptors” (DB00575) and two other calcium channel blockers, felodipine (DB01023) and nilvadipine (DB06712), which target five BP proteins each.

### BP-proteins and their association with diseases

Twenty-two of the 34 antihypertensive drug protein-targets, i.e., nine BNs (one prioritized), 6 GNs (three prioritized) and seven YNs (two prioritized), have been associated with complex diseases by OMIM, including diabetes mellitus, insulin resistance, obesity, myocardial infarction, vascular abnormalities, heart failure associated syndromes, ischemic stroke and Alzheimer’s disease (Additional file 4). The proteins associated with the highest number of OMIM disease IDs are ΑCE (BN; 8), PPARG (YN; 8), MTHR (GN; 7) and KCJ11 (BN; 6).

According to the Genetic Association Database (GAD) Resource (DAVID version), 78% of all the identified as BP-associated proteins have been related to diseases. About 80% of the disease-related BP-proteins have statistically significant association (*q*-value < 0.05) with at least one of 100 GAD terms in total (Additional file 4), including five terms directly associated with BP, i.e., “hypertension”, “blood pressure”, “blood pressure arterial”, “diastolic blood pressure” and “systolic blood pressure”. These five GAD terms are linked to 251 identified as BP-proteins from our analysis, i.e., 101 BNs (27 prioritized), 51 GNs (15 prioritized), 87 YNs (16 prioritized), 10 proteins (3 prioritized) with PPIs of low experimental confidence of being direct and 2 proteins (1 prioritized) with no known PPIs (Additional files 4, 5). These observations further support the validity of the YNs and the proposed BP-protein prioritization scheme.

**Fig. 13:**
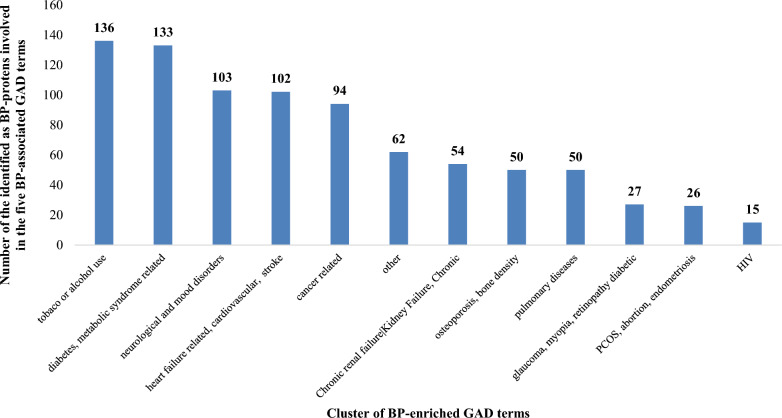
Distribution of BP-enriched GAD phenotype clusters regarding the number of included BP-proteins related to the BP-associated GAD terms

Six KEGG-defined pathways involve more than 20 proteins related with the five BP-associated GAD terms: cGMP-PKG signaling pathway (28), Pathways in Cancer (26), Metabolic Pathways (23), calcium signaling pathway (21), cAMP signaling pathway (21), PI3K-Akt signaling pathway (21). Searching for potential comorbidities, we grouped the rest 95 BP-protein-enriched GAD terms in 12 wider phenotype clusters and found that 89% of the 251 proteins linked to the five BP-related GAD term group have also been associated with at least one other phenotype than BP (Fig. 13, Additional file 4). Ninety-four (42%) are linked to at least four other phenotype clusters up to a maximum of 11 for five BP-proteins, i.e., TNFA (YN), APOE (BN), IGF1 (BN), ACE (BN), MTHR (GN, prioritized). The phenotype clusters that involve at least 100 BP-proteins linked to the five BP-related GAD terms are ‘tobacco or alcohol use’ (136), ‘diabetes, metabolic syndrome related’ (133), ‘neurological and mood disorders’ (103) and ‘heart failure related, cardiovascular, stroke’ (102).

## Discussion

### Introducing a BP-GWAS meta-database

In the present study, we implemented a BP-GWAS meta-database comprehensively collecting BP-associated data from both the GWAS Catalog database and by manual curation of the literature, with the latter subset constituting the majority of the retrieved GWAS data. The design of our meta-database (Fig. 2) enables for a GWAS study to be uniquely defined by a set of attributes. In this way, it is possible to distinctly store data from various studies reported in the same publication. Moreover, from the manually curated publications, we collected all significant variants associated with a particular gene locus. Similarly, if multiple BP-traits had been investigated in a GWAS, we collected all significant association *p*-values of a variant with any of the traits. In this way, our meta-database provides a comprehensive collection of all BP-associated GWAS data, enabling the querying of the dataset based on any combination of stored parameters. Thus, we can evaluate the significance of the BP-association of SNPs or gene loci based on a combination of attributes, strengthening the reliability of the suggested information and of relevant SNP or gene prioritization schemes.

Through the SNP genotypic information part of our meta-database, the recorded SNPs are linked to all their transcript consequences along with their Ensembl-defined severity score, widening the perspective of the BP-associated molecular physiology that can be extracted from GWAS. Our meta-database is designed to store locally eQTL measurement information, adding a supporting feature for the BP-association of the GWAS-identified SNPs and genes. At present, we have collected eQTL data from GTEx for 27 tissues considered to be involved in BP regulation. Through the human genetic information ontology network, one can now link the GWAS and the supporting eQTL data with any type of functional or omic data at various genetic information levels, including gene-disease and drug–protein associations, and collectively analyze the GWAS information based on combinations of GWAS attributes and biological criteria in the context of the associated biomolecular networks.

Among the main observations about GWAS-identified as BP-associated SNPs and genes enabled by the analysis of our comprehensive BP-GWAS data collection, we note: (i) the suggestion of a new stricter genome-wide significance threshold for SNP-BP trait associations identified in GWAS, i.e., 2.2 × 10^−12^, compared to the current generally considered 5 × 10^−8^ value, (ii) ~ 56% of the BP-SNPs are related to 1167 RHCP-coding genes, while 24% are intergenic, (iii) 665 of the 1167 RHCP-coding BP-GWAS genes are also supported by eQTLs in any of the 27 selected as BP-related tissues, and (iv) the most enriched in BP-GWAS genes human chromosomes are 1, 2 and 11. The genes *ATXN2* and *SH2B3*, which are at the core of the 12q24 chromosomal region, have been identified with the lowest BP-association *p*-value. The particular locus has been GWAS-associated with many diseases, including hypertension and cardiovascular infarction, along with autoimmune diseases, like diabetes 1 and hypothyroidism [60]. Loss of ATΧN2 function can lead to insulin resistance and obesity [61], while SH2B3, the SH2B adapter protein 3, has been causally associated with BP regulation [62].

### Introducing a new gene prioritization criterion based on an integrated GWAS score

Having all this information collected in a meta-database, we possess a valuable tool that enables the creation of BP-gene prioritization schemes based on integrated GWAS scores, taking into consideration more GWAS data attributes in addition to the minimum SNP/gene-trait association *p*-value, which has traditionally been the main prioritization criterion. These additional attributes include the number of significant SNPs per gene and the number of independent GWAS publications supporting the BP-association of a gene. In our newly proposed integrated BP-gene association score equaling the weighted sum of these three GWAS attributes, we still allocate the maximum significance to the *p*-value, assigning a corresponding weight of 45%, followed by a 35% weight for the number of SNPs per gene and a 20% weight for the number of independent publications, as the vast majority of the BP-genes are currently supported by at most two publications (Fig. 5). As GWAS evolve, the relative weight of the publication criterion could be increased and/or other criteria, including number of ancestries or eQTL measurements may also be considered in the combined score to enhance the validity of the prioritization scheme.

Our proposed GWAS data-based metric prioritized 103 RHCP-coding BP genes, of which 45 are mapped on chromosomes 1, 10, 11 and 12, and 69 are supported by eQTLs (Table 2, Additional file 1: Table S4, Additional file 3). The top-10 genes in decreasing score order are: *ULK4*, *ATP2B1*, *SH2B3*, *ATXN2*, *ZNF831*, *CNNM2, CLCN6*, *MTHFR*, *CABCOCO1* and *CSK* (Table 2). Among these, seven (*ULK4*, *ATP2B1*, *CNNM2*, *CLCN6*, *MTHFR*, *CABCOCO1*, *CSK*) are also supported by eQTLs in at least two of the BP-related tissues (Additional file 3). Notably, *ULK4* (unc-51 like kinase 4), *ZNF831* (zinc finger protein 831), *CNNM2* (cyclin and CBS domain divalent metal cation transport mediator 2) and *CABCOCO1* (ciliary-associated calcium binding coiled-coil 1), would not have been in the top-10 if only the BP-association *p*-value had been considered, but emerge as prioritized based on the number of significant SNPs and/or the number of independent publications (Table 2). We underline the BP-association of *ULK4*, which is supported by a very high number of SNPs, independent publications and eQTL measurements in 25 of the 27 BP-related tissues, and was first identified by early GWAS studies [63, 64]. Even though ULK4 has been traditionally considered as BP-associated based on GWAS, as also documented by its inclusion in the BP-associated protein set of GAD, its biological role remains unclear. There has been evidence that ULK4, a serine/threonine kinase, is responsible for over 90% of total Ser/Thr dephosphorylation in eukaryotes [65]. Through its interaction with the two most abundant phosphatases PP2A and PP1α, ULK4 regulates the expression of p-Akt and p-GSK-3α/β and may be involved in the remodeling of cytoskeletal components, participating in the regulation of neurite elongation and cell motility. *ULK4* has been proposed to be a rare susceptibility gene for psychiatric disorders, especially schizophrenia [65, 66]*.* The plasma membrane calcium-transporting ATPases as ATP2B1 play a major role in maintaining intracellular calcium homeostasis [67], being thus directly associated with BP regulation. Deficiency of the divalent cation metal transporter CNNM2 has been causally associated with hypomagnesemia and BP deregulation [68]. CLCN6, the transmembrane chloride transport protein 6, has been for long associated with BP through GWAS, but the actual mechanism has been recently elucidated as its inactivation is associated with arterial stiffness and alterations of vascular smooth muscle contractility by changing calcium concentration in the Golgi apparatus [69]. Methylenetetrahydrofolate reductase, encoded by *MTHFR*, is the enzyme catalyzing the biosynthesis of folate, the homocysteine co-substrate in its conversion to methionine, essential in keeping the homocysteine-methionine balance [70]. Its loss leads to increase in serum homocysteine, which has been associated with premature coronary disease [71] and cardiovascular risks in general [70]. MTHFR is an antihypertensive drug target. CSK suppresses the activity of Src-family kinases (SRKs) [72], and has been indicated as a key modulator of BP by influencing aldosterone production in adrenal gland [73] and vascular remodeling [74].

### Reconstructing a protein interactome of BP regulation through a newly proposed method

Α major objective of our study was to upgrade the information content of the BP-GWAS data by investigating their relationship and interconnectivity in the context of the human protein interactome. To this end, we mapped the protein products of the GWAS-identified as BP-related genes on the human PPI network and observed that 91% are nodes of the network (Fig. 8A; Additional file 4). About two-thirds of the network proteins form a large interconnected component and were color-coded and named “blue nodes” (BNs), with the rest called “green nodes” (GNs). The top 1% BN protein nodes with respect to their degree in the BN network are P53, UBC9, ESR1 (GWAS-prioritized), FYN, HDAC4, SMAD3 and STAT3 (Additional file 4). Cellular tumor antigen p53 (P53), along with HIF-1A/2A, have been associated with pulmonary hypertension [75] and demonstrated to have a regulatory role in cardiovascular pathophysiology [76, 77]. UBC9-mediated sumoylation has been associated with good cardiac function and efficient protein quality control in cardiomyocytes [78, 79]. The role of estrogen receptor (ESR1) in BP and cardiac pathophysiology has been largely acknowledged in recent years through combination of available evidence in various studies [80–82]. In cardiomyocytes, the SFK FYN has been identified as a negative feedback regulator of the GWAS-prioritized NADPH oxidase 4, NOX4 (GN), which produces ROS, with FYN expression being substantially decreased in failing human hearts [83]. The network-based elucidation of both FYN and STAT3 as BP-significant proteins is functionally supported, as the SFKs inhibit the STAT3 signaling, playing thus a significant role in vascular remodeling and pulmonary arterial vasoconstriction [84]. STAT3 has been largely discussed for its cardioprotective role [85, 86]. Histone deacetylase 4, HDAC4, has been identified as a crucial regulator of cardiac function [87], mediating vascular inflammation involved in the pathophysiology of hypertension [88], and has been recognized to play a pivotal role in myocardial ischemia–reperfusion injury [89]. Finally, SMAD3, an intracellular signal transducer and transcriptional modulator, has been implicated in pulmonary arterial hypertension through vascular remodeling [90], cardiac fibrosis [91] and renal inflammation and fibrosis [92].

Considering that the GNs should be closely related to the BNs as both protein-sets are BP-associated based on GWAS, we proposed a new method of extending the GWAS-identified BP PPI network through the shortest interaction paths bridging GNs to BNs into one connected component. The “reconstructed by shortest path” (RbSP) BP PPI network (Fig. 8B, Additional file 1: Fig. S2A; Additional file 4) comprises 15% and 17%, respectively, of the protein nodes and the interactions of the human interactome, including 62 of its 65 proteins with more than 300 interactions, underlying that BP regulation involves a large number of pathways of human physiology. Indeed, the BP-related network confirms the close relationship between BNs and GNs as 98% of GNs are second neighbors to a BN. Furthermore, this observation strongly supports the BP-association of the “in silico” identified as BP-related shortest-path intermediates, named “yellow nodes” (YNs), as the vast majority of YNs are common neighbors of the experimentally identified as BP-related BNs and GNs (Fig. 8B).

### Pathway-enrichment analysis and the role of the network-identified as BP-related proteins (YNs)

The in silico identified ΥΝs extend the knowledge about BP regulation beyond the experimentally identified by GWAS. Any functional validation of the BP-association of YNs could further support our shortest-path approach to extend the BP PPI network beyond the GWAS data. First, YNs enhance the acquired information about BP-related pathways as their presence substantially increases the number of functional KEGG-defined pathways that are identified as significantly BP-protein enriched; 87 compared to 26 pathways when only GWAS proteins are considered. Most of the 87 pathways (Table 6) have been strongly associated with BP regulation and/or heart pathophysiology through functional studies, including the three cardiomyopathy-associated pathways, i.e., dilated cardiomyopathy (DCM) (Additional file 1: Fig. S6), hypertrophic cardiomyopathy (HCM), and arrhythmogenic right ventricular cardiomyopathy (ARVC), the adrenergic signaling in cardiomyocytes (Fig. 10) [55, 93, 94], the hypoxia-induced factor-1 (HIF-1) pathway (Fig. 11) [95–99], the calcium signaling [100–102], the thyroid hormone signaling [103–105], the renin–angiotensin–aldosterone system/RAAS [106, 107], the insulin secretion and resistance [108–111] and the vascular smooth muscle contraction [112–114]. Notably, the main protein of the HIF-1 pathway, HIF-1A, is itself a YN and the pathway would not have been identified as significant, if the BP PPI network had not been accordingly extended by the shortest-path approach.

Other functionally BP-associated signaling pathways that emerged as significantly BP-enriched based on the RbSP PPI network node-set, include: the phosphoinositide-3-kinase (PI3K)–protein kinase B (PKB/Akt) signaling pathway [115–118], the cyclic adenosine monophosphate (cAMP) signaling pathway [119, 120] and the guanosine monophosphate (cGMP)-protein kinase G (PKG) signaling pathway (Additional file 1: Fig. S5) [54, 121]. These pathways encompass or interconnect with pathways that have been directly associated with BP, such as the adrenergic signaling in cardiomyocytes (Fig. 10), the calcium signaling pathway, the vascular smooth muscle contraction and the DCM pathway (Additional file 1: Fig. S6). Notably, the cGMP-PKG signaling, the calcium signaling, the cAMP signaling and the renin secretion pathways contain the highest number of antihypertensive drug targets (Table 7).

Crucial proteins in the aforementioned BP-related pathways are network-identified YNs. This observation adds to the validity of the PPI network analysis and our proposed way of extending the GWAS-deduced PPI network of BP and identifying the YNs. To support this last argument, we point out some characteristic examples of YNs with key role in BP-associated pathways. In the PI3K/AKT pathway, PI3K can be activated by multiple signals, including receptor tyrosine kinases e.g., EGFR (ΥΝ), INSR (BN), ERBB2 (YN), ERBB3 (YN) and IGF1R (YN) and cellular matrix components, leading to the activation of serine/threonine kinase AKT, including the isoforms AKT1 (YN) and AKT2 (BN). AKT regulates the activation of downstream targets, such as mTOR (YN), GSK-3 (isoform GSK3B is a YN) and NOS (isoform NOS3 is a BN) [117]. m-TOR [122], GSK-3 [123] and NOS [124, 125] play a major role in cardiovascular homeostasis and any deregulation could lead to heart failure.

Regarding the cAMP and cGMP signaling, both cAMP and cGMP are major regulators of cardiac function, contractility, and integrity [126]. cAMP, as the main second messenger of beta-adrenergic receptor signaling, is formed in response to G protein-coupled receptors as ADRB1 (GN) and ADRB2 (YN) [119, 120]. Notably, ADRB1 and ADRB2, along with the ADRAs, ADA1A (YN) and ADA2A (YN), are targets of a large number of antihypertensive drugs (Fig. 12A; Fig. 10C; Additional file 1: Figs. S5C, S6C; Additional file 4). The cAMP response element-binding (CREB)-binding protein (CBP; YN) and its closely related paralog EP300 (YN) have been indicated to participate in vascular smooth muscle contraction [127] and skeletal muscle homeostasis [128]. CBP and EP300 interact with many proteins, including, P53 (BN; GWAS-prioritized), HIF-1A (YN), JUN (YN), FOS (YN), TYY1 (BN), TF65 (BN) and steroid receptors, including ESR1 (BN; GWAS-prioritized), glucocorticoid receptor (GCR; YN) and androgen receptor (ANDR; YN) [129]. JUN is an antihypertensive drug target (Additional file 4). cGMP is formed in response to NO and natriuretic peptides, including the atrial natriuretic factor ANF (*NPPA*) (BN) and the brain natriuretic factor ANFB (*NPPB*) (BN), and has been shown to modulate hypertension via different mechanisms, as vasorelaxation or renin reduction [54].

Pathway enrichment analysis underlined the association of BP regulation with well-functioning cell–cell junctions, including adherens junctions, gap junctions and focal adhesions, along with the strongly related to cell junctions Hippo signaling pathway. Deregulation of intercellular interactions have been implicated in vascular and cardiac-related diseases, as discussed in detail in relevant reviews, e.g., [130–134]. CTNB1, the catenin-beta 1 protein (YN), plays an important role in cell–cell junctions and is a key component of WNT signaling pathway [135]. Changes in the activity of the WNT/β-catenin signaling pathway [136–138] and Hippo pathway [139, 140] have been associated with heart diseases and hypertension. Suppression of the Hippo and WΝΤ signaling pathways mediated by the activation of EP300/p53 pathway has been associated with severe deregulation of the apical junction in ARVC [141]. From the lipid-modified WNT proteins, we encounter WNT3A (YN), WNT2B (GN; GWAS-prioritized) and WNT9A (GN) in the GWAS-RbSP PPI network. A recent review summarizes the interplay between the WNT/β-catenin signaling pathway and the renin-angiontensin system (RAS) with PPARG (YN), a crucial member of lipid metabolism and antihypertensive drug target [142]. The functionally associated with BP and/or cardiovascular physiology lipid metabolism was indeed revealed as BP-protein enriched based on the extended BP-protein set, with respect to the biosynthesis of unsaturated fatty acids [143–145], the elongation of fatty acids in mitochondria and the β-oxidation of fatty acids [146], along with the steroid hormone synthesis [147], and the purine/pyrimidine biosynthesis [148, 149] metabolic pathways (Additional file 1: Fig. S7B).

### Introducing two network-based criteria for BP-protein prioritization

As the pathway analysis of the RbSP PPI network revealed processes that are indeed functionally supported as BP-related, with many of their crucial nodes being in silico identified YNs, we could trust the BP-association of the extended network and search for BP-protein prioritization criteria in the network metrics. Regarding the BP-relation of A4 (YN), the most connected node of the GWAS-RbSP PPI network, recent studies have established association between the progression of Alzheimer-like pathology and hypertension [150, 151]. EGFR (YN) and EP300 (YN), which are among the RbSP PPI network hubs with more than 200 interactions are documented as BP-related by their involvement in the PI3K-Akt, cAMP, HIF-1A, and the calcium, thyroid hormone, Hippo, and WNT signaling pathways (Additional file 4).

In this study, we opted to analyze the role of each protein-node in the RbSP BP interactome and use this information to develop a prioritization criterion, based on an integrated network metric, IVI, taking into consideration additional node characteristics beyond the number of interactions (degree). The top-10 of the 106 IVI-prioritized proteins are P53 (BN, BN network hub), UBC (YN), ESR1 (BN, GWAS-prioritized, BN network hub), EP300 (YN), A4 (YN), EGFR (YN), AKT1 (YN), BRCA1 (YN), CBP (YN) and heat shock protein HSP 90-alpha (HS90A, YN) (Table 3). As mainly expected from the IVI metric definition, all but CBP belong to the top-21 of the degree distribution. It is the high IVI-spreading index of CBP that contributes to its IVI being in the top-10. We have already discussed that CBP, a protein-lysine acetyltransferase, interacts with many proteins as a major component of the cAMP pathway. The role of the heat shock proteins in general, and the HSA90 molecular chaperone family, in particular, in cardiac homeostasis has been demonstrated through multiple studies, e.g., [152–157]. HSP90A interacts with many proteins including AKT1 (YN), AKT2 (BN), ANDR (YN), NOS3 (BN, GWAS-prioritized), GSK3B (YN), STAT3 (ΒN, ΒΝ network hub), P53 (BN) and HIF1A (YN), which have already been discussed as associated with cardiovascular pathophysiologies and/or as members of BP-related pathways. The role of BRCA1 in BP has been argued in the context of the high cardiovascular disease risk of BRCA1/2 mutation carriers [158] and the comorbidity of hypertension and breast cancer [159]. Other prioritized proteins that rank high in the IVI score distribution because of high spreading indices include SP1 (YN), HIF1A (YN) and TF65 (BN). SP1, a general transcription factor, is involved in the regulation of sarcoplasmic reticulum Ca^2+^-ATPase (SERCA) pump in cardiomyocytes [160, 161].

Interestingly, most of the BNs, including TF65 (alternatively, NF-kappa B p65 subunit) and the BN network hubs UBC9, SMAD3 and MAPK1 (or ERK2), which are prioritized based on the IVI metric (19 of 22 in total), have a low ranking in the integrated GWAS score list, an observation further supporting the value of the network-based analysis of the GWAS data. ESR1 is the only prioritized based on both criteria (Table 3, Additional file 5), while the IVI-prioritized BNs, P53 and IKBA rank slightly below the significance threshold of the GWAS-based prioritization criterion. The NF-κB signaling pathway has been linked to several heart pathologic processes [162]. TF65, in particular, promotes apoptosis in heart failure [163] and is required for the pressure overload compensation by cardiomyocytes; in its absence, cardiomyocytes fail to increase the expression of HIF1A (YN), the TF65 target protein [164]. Finally, the role of MAPKs in heart failure has been long known, e.g., [165–167].

Apart from the overall network metrics, as the degree or the IVI, we propose a novel network-based method to prioritize the in silico identified YNs, while still taking advantage of the information derived from the integrated GWAS-based prioritization scheme. Specifically, we assigned higher BP-relevance to the YNs that are common neighbors of GWAS-prioritized BNs and GNs, while the GWAS proteins gain additional credit from their involvement in this network. The 253 protein nodes identified in this connected subnetwork of the RbSP BP interactome were IVI-ranked (Table 4; Additional file 1: Fig. S3) and the top ten (all IVI-prioritized) proteins are: ESR1 (BN, GWAS- and IVI- prioritized, BN and RbSP PPI network hub), AKT1 (YN), EGFR (YN, RbSP PPI network hub), CTNB1 (YN), UBC (YN, RbSP PPI network hub), BRCA1 (YN), GRB2 (YN), A4 (YN, RbSP PPI network hub), SRC (YN) and EP300 (YN, RbSP PPI network hub). From the two proteins not in the top-10 of the IVI-prioritized, GRB2 is essential for cardiac hypertrophy upon pressure overload [168] and atherosclerotic cell formation [169], while it ,also, induces cardiorenal syndrome type 3 [170]. SRC is required for mechanical stress (MS)-induced cardiomyocyte hypertrophy [171] and activates various signaling pathways involved in cardiovascular diseases [172, 173].

### Introducing an integrated BP-protein prioritization scheme

In summary, the analysis of the collected BP-GWAS meta-dataset in the context of the human PPI network extended by the YNs enabled us to define one GWAS-based and two network-based criteria for gene/protein prioritization with respect to their association with BP and determine three respective BP-significant protein sets (Fig. 1). The union of the three sets is proposed as the complete set of prioritized BP-proteins (Additional file 1: Fig. S4), ranked based on the number of the satisfied prioritization criteria, formulating thus, an integrated BP-protein prioritization scheme (Additional file 5).

Notably, the only protein satisfying all three criteria is ESR1, strongly supporting its BP-association, with this observation emerging as a major result of our study. ESR1 has a very influential role in the BP RbSP PPI network, as it ranks very high in the network-based criteria (Tables 3 and 4), while it is only in position 51 in the GWAS-prioritized list. ESR1 is one of the three predominant estrogen receptors, and has been long known to protect against hypertension [174, 175]. Nine GWAS-prioritized BNs (INSR, PTN11, CDK6, CSK, NOS3, SH2B3, ATP2B1, FES and FINC) complete the top-10 BP-significant protein-set (Table 5, Additional file 5). The significant role of insulin receptors in cardioprotection has been demonstrated through the activation of the PI3K-AKT and the Ras-MAPK signaling pathways [176], while the first genetic variation associated with essential hypertension was in INSR [177]. Deletion of *PTPN11* has been shown to cause DCM, through loss of MAPK signaling pathway activation [178], while mutations of this gene have been associated with cardiac defects and insulin resistance [179]. CDK6, mainly discussed as an anti-cancer drug target, has been implicated in pulmonary arterial hypertension [180], loss of its activity can lead to heart failure [181] and is a major regulator of atherosclerosis [182]. FES has been shown to play a protective role against atherosclerosis [183]. Fibronectin (FINC, encoded by *FN1*) has a cardioprotective role, potentially through its contribution to the formation of a functional vascular wall extracellular matrix [184].

Overall, the integrated BP-prioritized protein set provides a valuable resource of proteins suggested as BP-significant according to quantitative criteria, which combine GWAS-based importance with the influential role of a protein in the topology of the PPI network, increasing thus the confidence in the validity of the prioritization. Interestingly, thyroid hormone and neurotrophin signaling pathways have been identified of higher enrichment in the BP-prioritized compared to the overall BP-protein set, further supporting the association of these pathways with BP. Thyroid hormones regulate mechanisms underlying hypertension [103, 185]. Neurotrophins, as the brain-derived neurotrophic factor (BDNF; BN), have been directly linked to hypertension through the regulation of angiotensin signaling [186, 187]. Recent studies causally implicate neurotrophins with Alzheimer’s and Huntington’s diseases [188]. Comorbidity analysis based on the BP-associated GAD ontology terms indicated higher comorbidity of BP deregulation with alcohol and tobacco use, diabetes and metabolic syndrome, neurological and mood disorders, cardiovascular diseases, cancer, and renal failure, e.g., [189–192].

## Conclusions

In this study, we introduced an integrated workflow for upgrading the information content of ΒP-GWAS data through PPI network analysis, starting from the development of a systematically curated BP-GWAS meta-database, combining GWAS data with their transcript effects and eQTL measurements, leading to their projection on the human PPI network. The information stored in the meta-database lead to the definition of an integrated GWAS-based prioritization criterion for BP-associated genes, considering not only the minimum SNP-trait *p*-value per gene, but also the number of BP-associated SNPs per gene and the number of independent supporting publications. The projection of the GWAS data on the human protein interaction network revealed connected and non-connected components, which we proposed to link through shortest paths (GWAS-RbSP PPI network). Thus, we introduced a novel extension method for GWAS-based disease-related PPI networks, considering the intermediate nodes of the shortest paths (YNs) as also related to the investigated phenotype. Pathway enrichment analysis of the RbSP PPI network revealed BP-enriched pathways, indicating underlying mechanisms and targets for drugs and therapeutic treatments, which were interpreted in the context of available functional information. The role of each protein-node in the RbSP PPI network based on network metrics provided a second BP-protein prioritization criterion. A third prioritization criterion proposed in this study revealed the YNs that are common neighbors of GWAS-prioritized proteins. The integrated BP-prioritization set was topped by the proteins satisfying at least two of the prioritization criteria, ESR1 emerging as the most BP-significant. This analysis extends our knowledge about BP regulation and could be effectively applied to GWAS datasets of any multifactorial disease. In the limitations of our study, we note that our results were obtained without making any distinction between the origin/ancestry profile of the cohorts to which the combined GWAS data referred. At the moment, such distinction is expected to be biased toward the European origin, as this represents the vast majority of the GWAS cohorts. As GWAS data from other ancestries increase, such distinction, which is accommodated by the structure of our meta-database could lead to useful ancestry-specific results about BP. Furthermore, there may be a bias in the significance of the number of SNPs per gene criterion for certain genes in the integrated GWAS score, due to dependencies in the reported SNPs. Finally, the selection of the weights of the three gene attributes in the integrated GWAS score has been carried out based on the current relevant distributions of the GWAS genes. The relative weights may be modified in the future as more GWAS publications and/or information about independent SNPs becomes available.

## Supplementary Information

**Additional file 1**. **Table S1**: The GWAS Catalog mapped traits associated with BP; **Table S2**: The 10 BP-associated EFO terms included in the GWAS Catalog mapped traits; **Table S3**: The curated BP-associated GWAS publications; **Table S4**: Chromosomal distribution of SNPs & associated RHCP-coding genes, including the prioritized genes by Criterion 1; **Table S5**: Categorization of the BP SNPs based on the type and number of transcript consequences. **Figure S1**: The PPI Network of BNs and its characteristics; **Figure S2**: The GWAS-RbSP PPI Network and its characteristics; **Figure S3**: The IVI-scaled PPI network of the GWAS-prioritized BP-proteins and their common YN neighbours; **Figure S4**: Venn diagram of the complete set of prioritized BP-proteins, regarding the three prioritization criteria; **Figure S5**: The BP-proteins in the KEGG-defined cGMP-PKG signaling pathway and their PPI network; **Figure S6**: The BP-proteins in the KEGG-defined dilated cardiomyopathy (DCM) pathway and their PPI network; **Figure S7**: The metabolic reactions catalyzed by BP-GWAS proteins (A) and RbSP PPI network proteins (B).

**Additional file 2**. The BP-associated RHCP-coding genes and their attributes.

**Additional file 3**. SNP-gene associations supported by cis-eQTL measurements in at least one of the 27 selected tissues.

**Additional file 4**. The BP-proteins in the GWAS-RbSP PPI network and their attributes.

**Additional file 5**. The overall and criterion-specific rankings of the prioritized BP-proteins.

## Data Availability

All data generated or analyzed during this study are included in this article and its supplementary information files.
