# Supplementary material for: Protein–protein interaction network-based integration of GWAS and functional data for blood pressure regulation analysis
Source: Hum Genomics. 2024 Feb 8;18:15. doi: 10.1186/s40246-023-00565-6 (PMC11465932; doi:10.1186/s40246-023-00565-6)
Supplement: Supplementary file 1 — Additional file 1. Table S1: The GWAS Catalog mapped traits associated with BP; Table S2: The 10 BP-associated EFO terms included in the GWAS Catalog mapped traits; Table S3: The curated BP-associated GWAS publications; Table S4: Chromosomal distribution of SNPs & associated RHCP-coding genes, including the prioritized genes by Criterion 1; Table S5: Categorization of the BP SNPs based on the type and number of transcript consequences. Figure S1: The PPI Network of BNs and its characteristics; Figure S2: The GWAS-RbSP PPI Network and its characteristics; Figure S3: The IVI-scaled PPI network of the GWAS-prioritized BP-proteins and their common YN neighbours; Figure S4: Venn diagram of the complete set of prioritized BP-proteins, regarding the three prioritization criteria; Figure S5: The BP-proteins in the KEGG-defined cGMP-PKG signaling pathway and their PPI network; Figure S6: The BP-proteins in the KEGG-defined dilated cardiomyopathy (DCM) pathway and their PPI network; Figure S7: The metabolic reactions catalyzed by BP-GWAS proteins (A) and RbSP PPI network proteins (B). [file 40246_2023_565_MOESM1_ESM.pdf]

Additional File 1

Table of Contents

Table S1: The GWAS Catalog mapped traits associated with BP 2

Table S2: The 10 BP-associated EFO terms included in the GWAS Catalog mapped traits 4

Table S3: The curated BP-associated GWAS publications 5

Table S4: Chromosomal distribution of SNPs & associated RHCP-coding genes, including the prioritized genes by Criterion 1 7

Table S5: Categorization of the BP SNPs based on the type and number of transcript consequences 8

Figure S1: The PPI Network of BNs and its characteristics. 9

Figure S2: The GWAS-RbSP PPI Network and its characteristics. 10

Figure S3: The IVI-scaled PPI network of the GWAS-prioritized BP-proteins and their common YN neighbours.. 11

Figure S4: Venn diagram of the complete set of prioritized BP-proteins regarding the three prioritization criteria. 12

Figure S5: The BP-proteins in the KEGG-defined cGMP-PKG signaling pathway and their PPI network. 13

Figure S6: The BP-proteins in the KEGG-defined dilated cardiomyopathy (DCM) pathway and their PPI network. 14

Figure S7: The metabolic reactions catalyzed by BP-GWAS proteins (A) and RbSP PPI network proteins (B). 15

Table S1: The GWAS Catalog mapped traits associated with BP

	**Mapped trait**
1.	blood pressure
2.	blood pressure, alcohol drinking
3.	blood pressure, chronic obstructive pulmonary disease
4.	blood pressure, response to thiazide, response to beta blocker
5.	diabetes mellitus, hypertension, medication adherence behavior
6.	diastolic blood pressure
7.	diastolic blood pressure change measurement
8.	diastolic blood pressure change measurement, hypertension
9.	diastolic blood pressure, alcohol drinking
10.	diastolic blood pressure, blood pressure, age at assessment
11.	diastolic blood pressure, body height
12.	diastolic blood pressure, cigarettes per day measurement
13.	diastolic blood pressure, response to cold pressor test
14.	diastolic blood pressure, response to high sodium diet
15.	diastolic blood pressure, response to high sodium diet, response to dietary potassium supplementation
16.	diastolic blood pressure, response to low sodium diet
17.	diastolic blood pressure, response to thiazide, response to beta blocker
18.	diastolic blood pressure, smoking status measurement
19.	early onset hypertension
20.	hypertension
21.	hypertension, cardiovascular disease, response to angiotensin-converting enzyme inhibitor
22.	hypertension, cardiovascular disease, response to calcium channel blocker
23.	hypertension, response to calcium channel blocker, myocardial infarction
24.	hypertension, short sleep
25.	mean arterial pressure
26.	mean arterial pressure, alcohol drinking
27.	mean arterial pressure, blood pressure, age at assessment
28.	mean arterial pressure, response to cold pressor test
29.	mean arterial pressure, response to high sodium diet
30.	mean arterial pressure, response to high sodium diet, response to dietary potassium supplementation
31.	mean arterial pressure, response to low sodium diet
32.	multiple sclerosis, systolic blood pressure
33.	pack-years measurement, diastolic blood pressure
34.	pack-years measurement, systolic blood pressure
35.	pulmonary arterial hypertension
36.	pulse pressure measurement
37.	pulse pressure measurement, alcohol drinking
38.	pulse pressure measurement, blood pressure, age at assessment
39.	pulse pressure measurement, diastolic blood pressure, systolic blood pressure, hypertension
40.	pulse pressure measurement, early onset hypertension
41.	response to antihypertensive drug, diabetes mellitus, hypertension, response to beta blocker, response to calcium channel blocker

42.	response to candesartan, hypertension
43.	response to hydrochlorothiazide, diastolic blood pressure change measurement, hypertension
44.	response to hydrochlorothiazide, hypertension, systolic blood pressure change measurement
45.	smoking behavior, smoking status measurement, systolic blood pressure
46.	sodium measurement, diastolic blood pressure
47.	sodium measurement, mean arterial pressure
48.	sodium measurement, systolic blood pressure
49.	systolic blood pressure
50.	systolic blood pressure change measurement
51.	systolic blood pressure change measurement, hypertension
52.	systolic blood pressure, alcohol drinking
53.	systolic blood pressure, body mass index
54.	systolic blood pressure, cigarettes per day measurement
55.	systolic blood pressure, dietary potassium intake measurement
56.	systolic blood pressure, response to cold pressor test
57.	systolic blood pressure, response to high sodium diet
58.	systolic blood pressure, response to high sodium diet, response to dietary potassium supplementation
59.	systolic blood pressure, response to low sodium diet
60.	systolic blood pressure, response to thiazide, response to beta blocker
61.	systolic blood pressure, smoking behavior measurement
62.	systolic blood pressure, smoking status measurement

Table S2: The 10 BP-associated EFO terms included in the GWAS Catalog mapped traits

EFO ID	EFO term
0000537	hypertension
0001361	pulmonary arterial hypertension
0004325	blood pressure
0004772	early onset hypertension
0005763	pulse pressure measurement
0006335	systolic blood pressure
0006336	diastolic blood pressure
0006340	mean arterial pressure
0006944	systolic blood pressure change measurement
0006945	diastolic blood pressure change measurement

Table S3: The curated BP-associated GWAS publications

PubMed ID [1] [2]	First Author	Publication date	GWAS Catalog curation date(s)	Retrieved SNP-trait associations with p-value < 10^{-5}
17554300	Welcome Trust Case Control Consortium	7/6/2007	16/6/2008	2
**17903302**	Levy D	19/9/2007	18/6/2008;12/9/2008	22
19114657	Wang Y	29/12/2008	14/1/2009	1
19304780	Org E	20/3/2009	2/4/2009	1
**19421330**	Yang HC	7/5/2009	21/5/2009	4
**19430479**	Levy D	10/5/2009	19/5/2009	18
**19430483**	Newton-Cheh C	10/5/2009	19/5/2009	9
19609347	Adeyemo A	17/7/2009	12/8/2009	2
**21082022**	Padmanabhan S	28/10/2010	16/12/2010	1
**21347282**	Lettre G	10/2/2011	13/5/2015	8
**21572416**	Kato N	15/5/2011	13/7/2011	17
**21626137**	Slavin TP	28/5/2011	16/6/2011	18
**21909110**	Wain LV	11/9/2011	11/10/2011	41
**21909115**	Ehret GB	11/9/2011	21/10/2011	50
**22322875**	Kim SJ	9/2/2012	3/3/2012	3
22384028	Guo Y	24/2/2012	20/3/2012	3
22566498	Turner ST	7/5/2012	21/7/2012	25
22763476	Zhang D	5/7/2012	12/9/2012	4
**23502781**	Germain M	17/3/2013	21/6/2013	1
**23972371**	Franceschini N	20/8/2013	15/3/2014	11
**24001895**	Kelly TN	3/9/2013	26/3/2014	6
**24165912**	He J	28/10/2013	26/4/2014	51
**24376456**	Simino J	12/12/2013	29/7/2014	28
24892410	Leu HB	3/6/2014	10/12/2014	2
**24903457**	Kim YK	5/6/2014	23/5/2015;12/9/2017	4
**24954895**	Simino J	19/6/2014	15/1/2015	37
**25189868**	Sung YJ	3/9/2014	23/6/2015	37
**25249183**	Lu X	23/9/2014	23/6/2015	41
25317111	Seo I	30/9/2014	29/5/2015	2
**26390057**	Kato N	21/9/2015	14/10/2016	61
26516778	Bis JC	30/10/2015	4/10/2016;6/10/2016	4
**26752167**	Taylor JY	11/1/2016	24/11/2016	8
**26920376**	Wang Y	26/2/2016	12/2/2019	3
**26969751**	Parmar PG	11/3/2016	24/2/2017	28
**27271309**	Li C	1/8/2016	16/8/2018	14
**27480026**	Liu X	2/8/2016	13/2/2017	1
**27618447**	Surendran P	1/10/2016	30/7/2018	397
**27618448**	Liu C	1/10/2016	7/9/2018	601
**27618452**	Ehret GB	12/9/2016	14/9/2018	156
27670767	Chang SW	27/9/2016	24/3/2017	3
**27736895**	Franceschini N	13/10/2016	28/2/2019	53
27802415	Salvi E	31/10/2016	24/3/2017	35
**27841878**	Hoffmann TJ	14/11/2016	10/2/2019	1414
**27980656**	Justice AE	18/10/2016	28/3/2017	10
**28135244**	Warren HR	30/1/2017	1/7/2017	234
**28234671**	Nandakumar P	1/7/2017	16/5/2019	9
**28273873**	Park YM	5/3/2017	6/6/2017	3
**28348047**	Li C	1/4/2017	29/6/2017	11
**28498854**	Liang J	12/5/2017	24/7/2017	157
**28739976**	Wain LV	24/7/2017	22/9/2017	212
28871152	Sofer T	4/9/2017	19/10/2017	2
**29030403**	Kraja AT	13/10/2017		117

**29088834**	Kimura M	24/8/2017	10/11/2017	1
29097388	Magvanjav O	2/11/2017	5/12/2017	6
29208002	Kim M	6/12/2017	17/1/2018	12
29212154	Lin E	16/9/2017	13/7/2018	4
**29212900**	Li C	6/12/2017	30/1/2018	1
**29403010**	Kanai M	5/2/2018	28/7/2018	90
**29455858**	Sung YJ	15/2/2018	5/9/2018	13716
**29615537**	Kulminski AM	1/3/2018	2/7/2018	5
**29912962**	Feitosa MF	18/6/2018	29/8/2018;18/10/2018	1546
**29973135**	Rimpela JM	4/7/2018	19/9/2018	14
**30224653**	Evangelou E	17/9/2018	29/11/2018	4055
**30242241**	Hachiya T	21/9/2018	11/2/2019	90
**30487518**	Takeuchi F	28/11/2018	2/5/2019	915
**30527956**	Rhodes CJ	5/12/2018	27/2/2019	14
**30578418**	Giri A	21/12/2018	7/3/2019	1321
**30595370**	Kichaev G	27/12/2018	8/2/2019	1658
**30940143**	Zhu Z	2/4/2019	18/4/2019	22
**31217584**	Wojcik GL	19/6/2019	2/7/2019	28
			**Total associations retrieved**	27480

[1] The PubMed IDs of publications from which at least one SNP-trait association with p-value < $5x10^{-8}$ was retrieved, are shown in bold.

[2] Manually curated publications are shown in gray background.

Table S4: Chromosomal distribution of SNPs & associated RHCP-coding genes, including the prioritized genes by Criterion 1

**Chromosome**	**Chromosome size (bp)**	**Total number of SNPs**	**Number of SNPs associated with RHCP-coding transcripts**	**Density of RHCP-associated SNPs (Number of SNPs/Mbp)**	**Number of RHCP-coding genes** (% fraction of the total)	**Number of RHCP UniProt IDs** [1]	**Number of GWAS Prioritized Genes** (% fraction of the RHCP-coding gene set) *Criterion 1*
1	248,956,422	595	431	1.7	112 (9.6%)	112	10 (8.9 %)
2	242,193,529	396	179	0.7	86 (7.4%)	87	4 (4.7 %)
3	198,295,559	676	535	2.7	69 (5.9%)	69	5 (7.2 %)
4	190,214,555	193	94	0.5	46 (3.9%)	46	6 (13 %)
5	181,538,259	443	90	0.5	47 (4%)	47	4 (8.5 %)
6	170,805,979	553	164	1.0	76 (6.5%)	76	7 (9.2 %)
7	159,345,973	199	102	0.6	63 (5.4%)	63	3 (4.8 %)
8	145,138,636	700	326	2.2	53 (4.5%)	53	7 (13.2 %)
9	138,394,717	97	56	0.4	34 (2.9%)	34	1 (2.9%)
10	133,797,422	462	351	2.6	72 (6.2%)	72	12 (16.7 %)
11	135,086,622	483	287	2.1	83 (7.1%)	83	12 (14.5 %)
12	133,275,309	483	225	1.7	76 (6.5%)	76	11 (14.5 %)
13	114,364,328	66	30	0.3	20 (1.7%)	20	-
14	107,043,718	91	56	0.5	39 (3.3%)	39	1 (2.6 %)
15	101,991,189	336	187	1.8	55 (4.7%)	55	6 (10.9 %)
16	90,338,345	247	189	2.1	48 (4.1%)	48	5 (10.4 %)
17	83,257,441	170	99	1.2	67 (5.7%)	67	2 (3%)
18	80,373,285	66	37	0.5	21 (1.8%)	21	1 (4.8 %)
19	58,617,616	121	85	1.5	46 (3.9%)	46	3 (6.5 %)
20	64,444,167	231	167	2.6	26 (2.2%)	28	2 (7.7 %)
21	46,709,983	35	16	0.3	12 (1%)	12	-
22	50,818,468	42	32	0.6	16 (1.4%)	16	1 (6.3 %)
X	156,040,895	2	-	-	-	-	-
Y	57,227,415	-	-	-	-	-	-
ALL		**6687**	**3738**		**1167**	**1170**	**103 (8.8%)**

[1] *GNAS* mapped to chromosome 20 corresponds to three UniProt IDs; *NRXN1* mapped to chromosome 2 corresponds to two UniProt IDs

Table S5: Categorization of the BP SNPs based on the type and number of transcript consequences

		SNP Category Description	# SNPs	# Coding Transcript Consequences	# of RHCP-coding Transcript Consequences	# RHCP Gene IDs	# RHCP UniProt_IDs
SNP Category with Respect to Transcript Consequence	A	One SNP to one RHCP-coding transcript consequence and vice versa	112	112	112	112	112
	B	One SNP to many (including RHCP-) coding transcript consequences	554	3390	1759	559	561
	C	Many SNPs to one RHCP-coding transcript consequence	138	47	47	65	47
	D	Many SNPs to many (including RHCP-) coding transcript consequences	2934	2726	1311	449	450
	E	SNPs with only non-RHCP coding transcript consequences	288 [1]	181 [47 common with the Category D coding transcripts]	-	-	-
	F	SNPs with only non-coding transcript consequence(s)	1082	-	-	-	-
	G	intergenic	1579	-	-	-	-
		**Total**	**6687**	**6409**	**3229**	**1167**	**1170**

[1] Categorization of SNPs to non-RHCP coding transcripts: 1-1 (33 SNPs/transcripts); 1-many (15 SNPs to 46 transcripts); many to 1 (80 SNPs to 20 transcripts); many to many (160 SNPs to 82 transcripts)

.

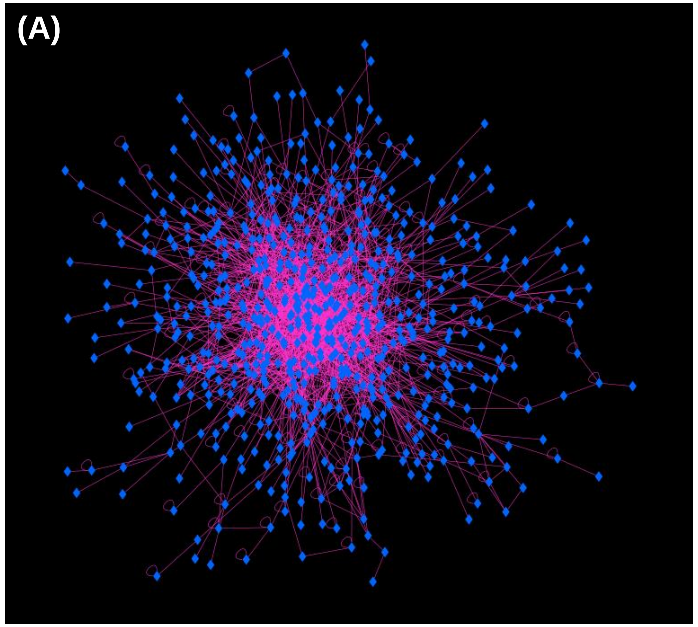

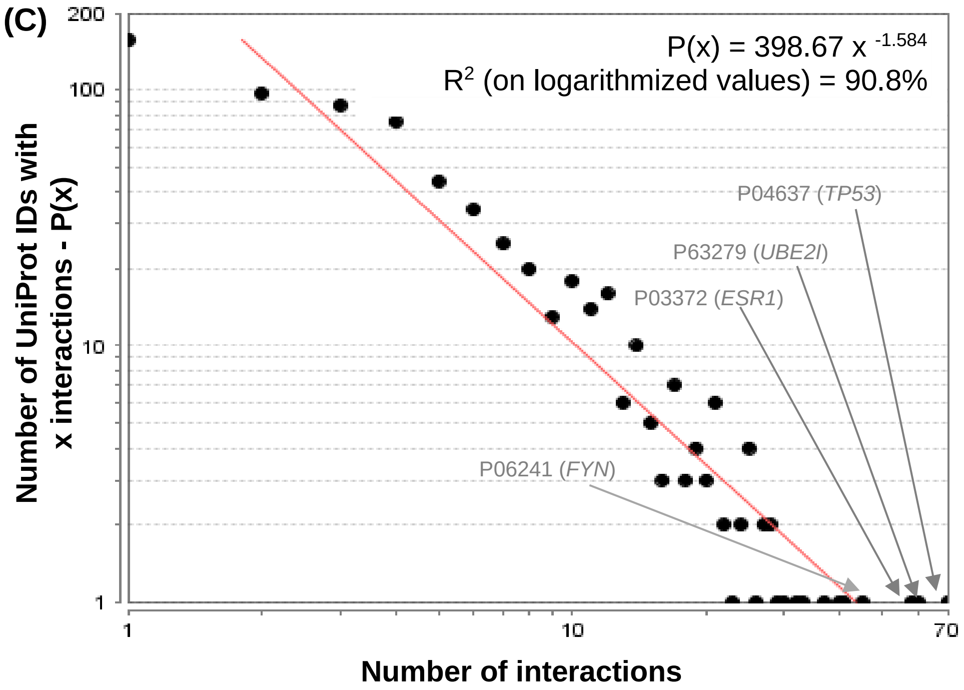

(B)

Network radius/diameter	6/10
Characteristic Path Length	3.868
Maximum/ Average Number of Neighbors	69/5.060
Clustering Coefficient	0.098
Network Density	0.008

Figure S1: The PPI Network of BNs and its characteristics. The force-directed representation (A), the network statistics (B), and the degree distribution and power-law fit (C). The BNs with the highest degree are indicated in (C).

.

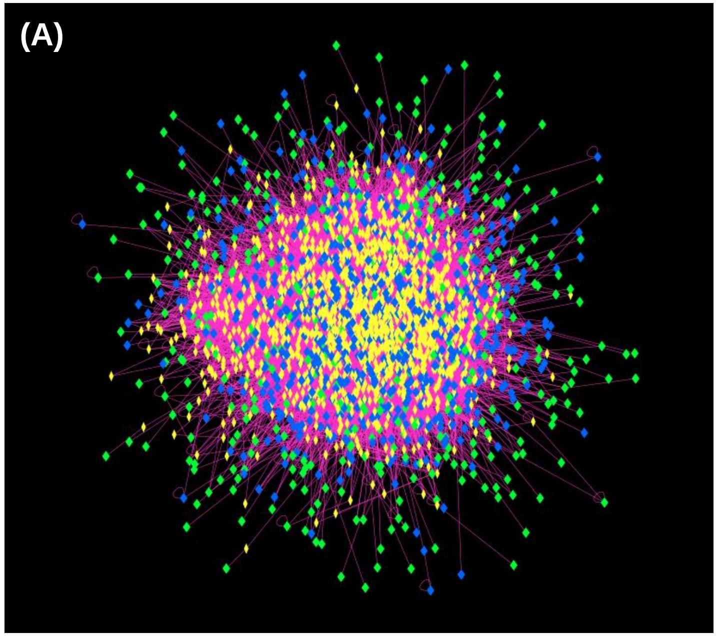

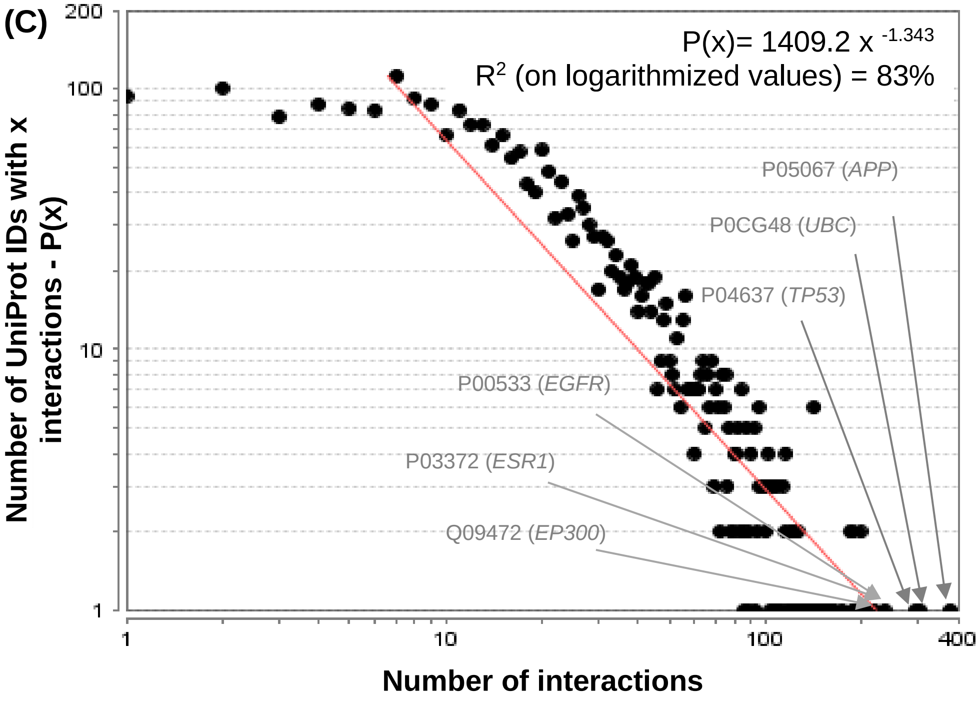

(B)

Network radius/diameter	4/7
Characteristic Path Length	3.868
Maximum/ Average Number of Neighbors	377/25.071
Clustering Coefficient	0.133
Network Density	0.010

Figure S2: The GWAS-RbSP PPI Network and its characteristics. The force-directed representation (A), the network statistics (B), and the degree distribution and power-law fit (C). Nodes in (A) are colour-coded as described in Figure 8. The nodes with the highest degree are indicated in (C).

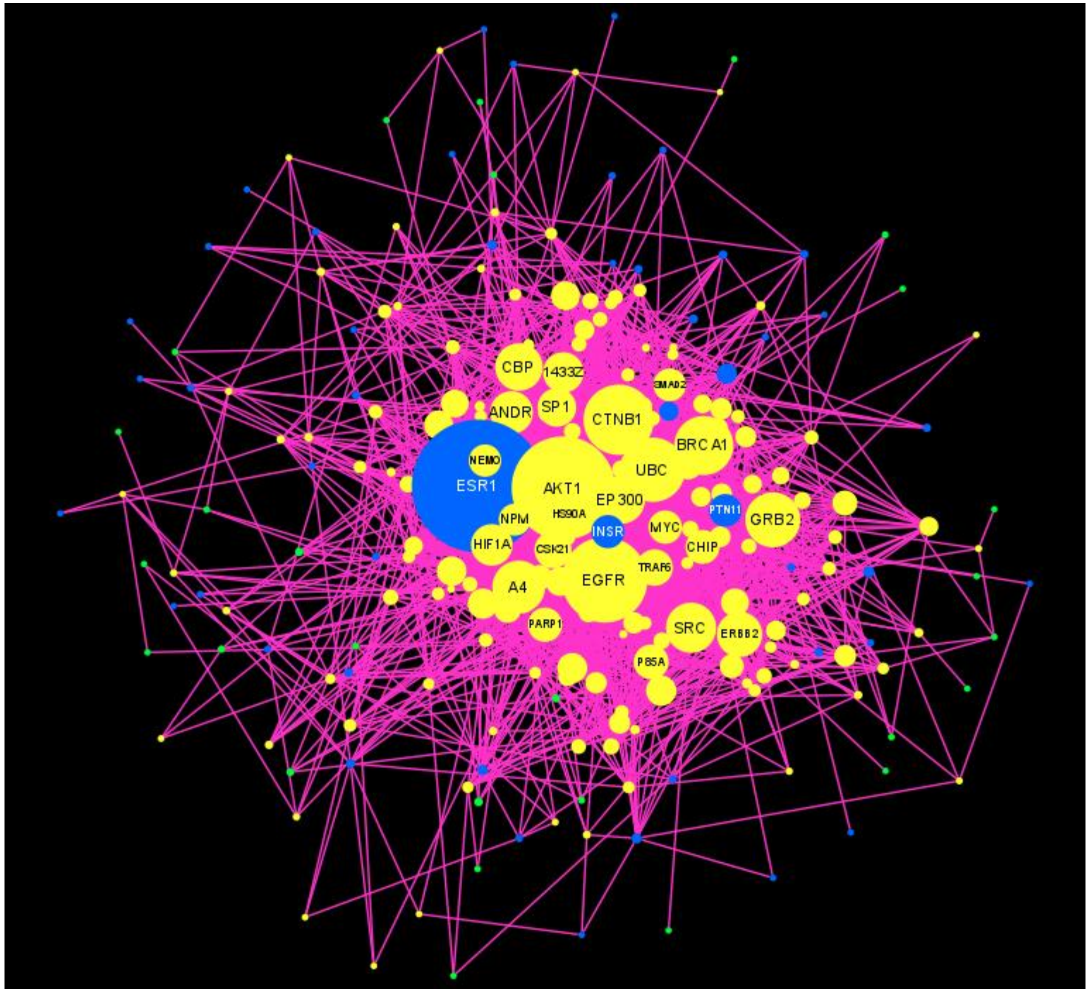

Figure S3: The IVI-scaled PPI network of the GWAS-prioritized BP-proteins and their common YN neighbours. Nodes are colour-coded as explained in Figure 8 and their size is scaled based on their IVI in this network. The UniProt Entry Names (excluding the _HUMAN extension) of the protein-nodes with IVI > 20 are shown.

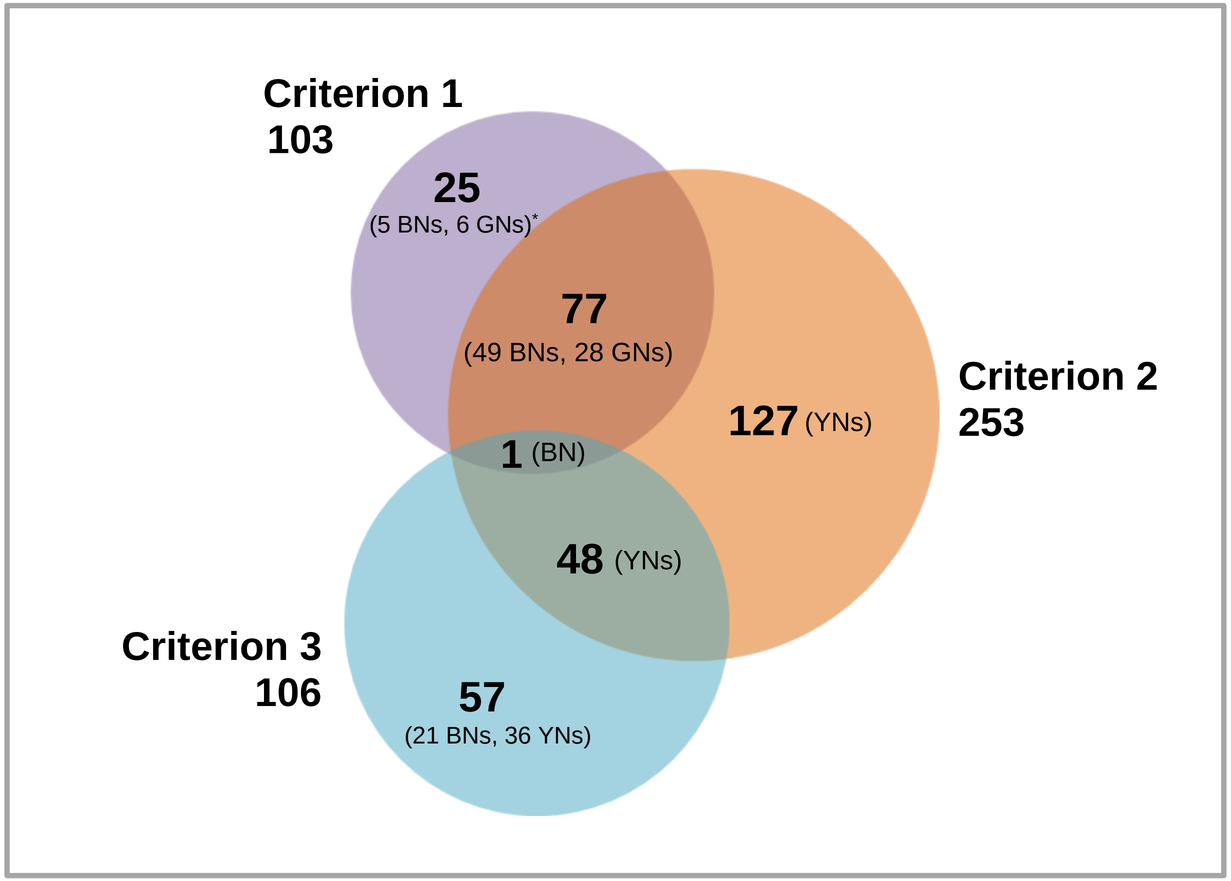

Figure S4: Venn diagram of the complete set of prioritized BP-proteins regarding the three prioritization criteria. Criterion 1: Integrated GWAS score; Criterion 2: IVI significance threshold in the GWAS-RbSP BP PPI network; Criterion 3: inclusion in the network of the GWAS-prioritized BP proteins and their common YNs. BN, GN, YN denote, respectively, the blue, green and yellow nodes with the colour-code explained in Figure 8. (*) The unique subset of the prioritized proteins based on Criterion 1 includes also 10 proteins with PPIs of low experimental confidence of being direct (unfiltered PICKLE PPI network) and 4 proteins with no known PPIs.

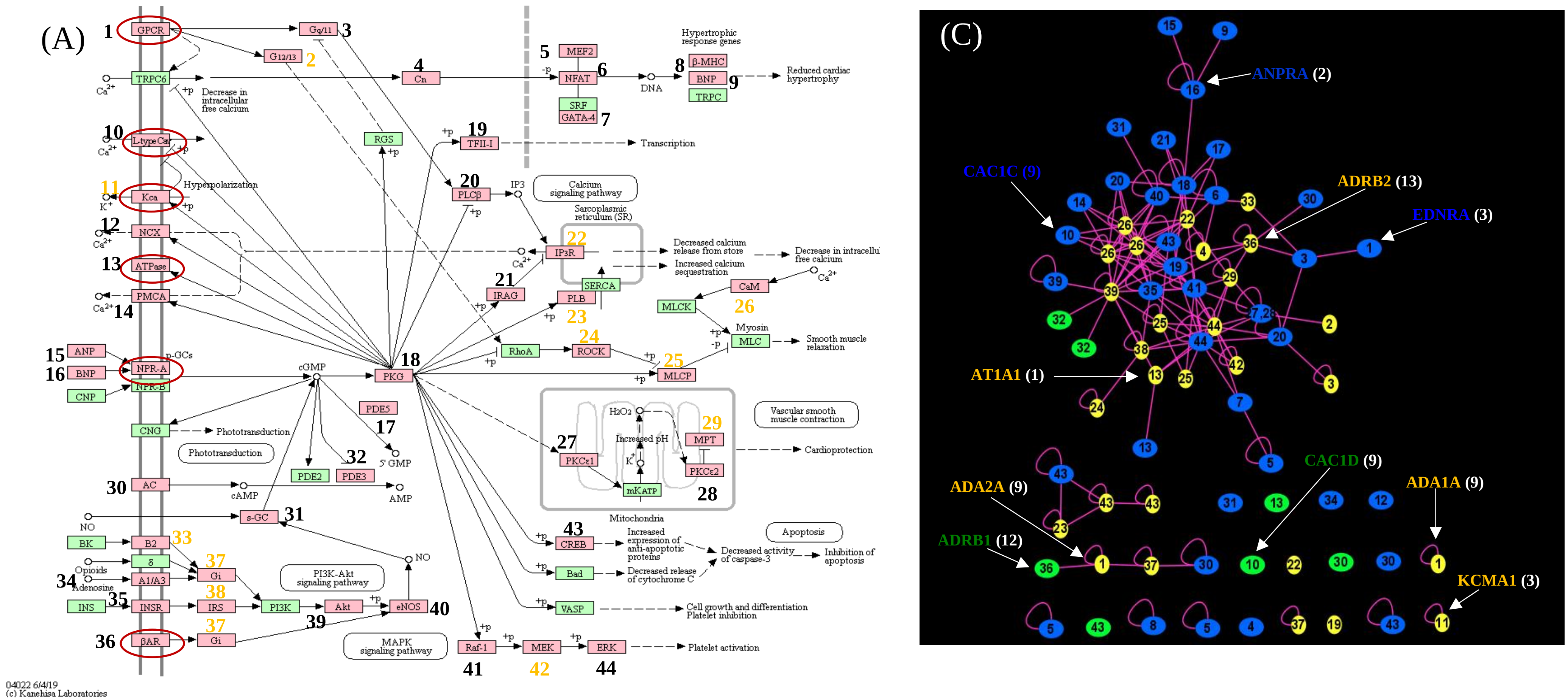

(B) 39% YNs

No.	Node	Gene symbols
1	GPCR	*ADRA1A*, *ADRA2A*, *EDNRA*
2	G12/13	*GNA13*
3	Gq/11	*GNAQ*, *GNA11*
4	Cn	*PPP3CA*, *PPP3CC*
5	MEF2	*MEF2A*, *MEF2B*, *MEF2C*
6	NFAT	*NFATC2*
7	GATA-4	*GATA4*
8	β-MHC	*MYH6**
9	BNP	*NPPB*
10	Ltype Ca	*CACNA1C*, *CACNA1D**
11	Kca	*KCNMA1*
12	NCX	*SLC8A1*
13	ATPase	*ATP1A1*, *ATP1B1*, *ATP1B3*
14	PMCA	*ATP2B1**
15	ANP	*NPPA**
16	NPR-A	*NPR1*
17	PDE5	*PDE5A*
18	PKG	*PRKG1*
19	TFII-I	*GTF2I*, *GTF2IRD1*
20	PLCβ	*PLCB1*, *PLCB3*
21	IRAG	*MRVI1*
22	IP₃R	*ITPR1*, *ITPR2*
23	PLB	*PLN*
24	ROCK	*ROCK1*
25	MLCP	*PPP1CA**, *PPP1CC**
26	CaM	*CALM1**, *CALM2**, *CALM3**
27	PKCε1	*PRKCE*
28	PKCε2	*PRKCE*
29	MPT	*VDAC1*
30	AC	*ADCY3*, *ADCY4* *ADCY5*, *ADCY9*
31	s-GC	*GUCY1A1**, *GUCY1B1*
32	PDE3	*PDE3A*, *PDE3B*
33	B2	*BDKRB2**
34	A1/A3	*ADORA1*
35	INSR	*INSR**
36	βAR	*ADRB1**, *ADRB2**
37	Gi	*GNAI1*, *GNAI3*
38	IRS	*IRS1**
39	Akt	*AKT1**, *AKT2*
40	eNOS	*NOS3**
41	Raf-1	*RAF1**
42	MEK	*MAP2K1*
43	CREB	*CREB1*, *CREB3*, *CREB3L1*, *CREB5*, *CREB3L2*, *ATF4*
44	ERK	*MAPK1**, *MAPK3**

Figure S5: The BP-proteins in the KEGG-defined cGMP-PKG signaling pathway and their PPI network. The BP proteins are shown (A) in the context of the pathway map, (B) with their gene symbols in respect to the numbered pathway map node symbols, and (C) in their subnetwork of the GWAS-RbSP protein interactome. BP-proteins in (C) are denoted by the number of the associated pathway map node. The figure is colour-coded and structured as described in the legend of Figure 10.

(A)

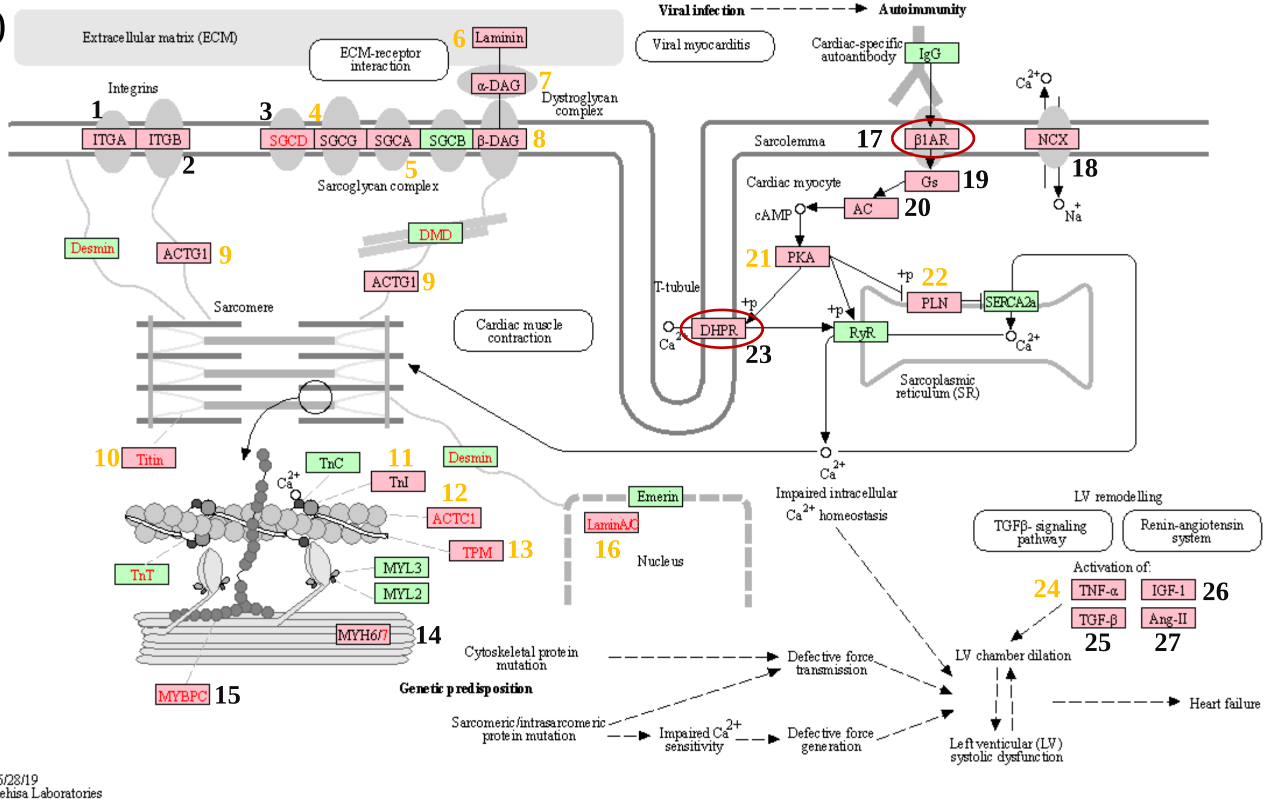

(C)

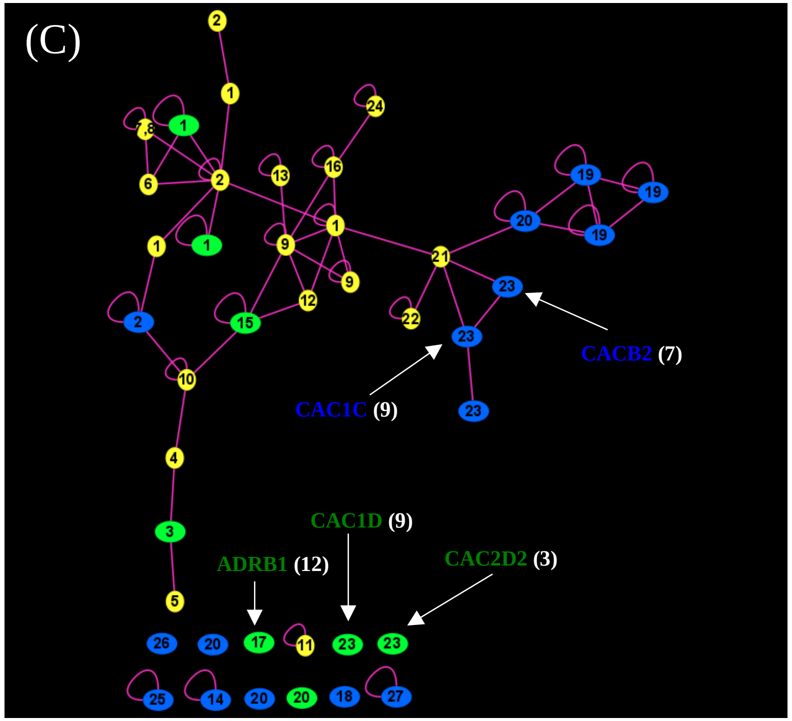

(B) 44% YNs

1	ITGA	*ITGA1*, *ITGA4**, *ITGA5*, *ITGA6*, *ITGA9*, *ITGA11*	6	Laminin	*LAMA1*	11	TnI	*TNNI3*	16	LaminA/C	*LMNA**	21	PKA	*PRKACA**	26	IGF-1	*IGF1*
2	ITGB	*ITGB1*, *ITGB4*, *ITGB5*	7	α-DAG	*DAG1*	12	ACTC1	ACTC1	17	β1-AR	*ADRB1**	22	PLN	*PLN*	27	Ang-II	*AGT**
3	SGCD	*SGCD*	8	β-DAG	*DAG1*	13	TPM	*TPM3*	18	NCX	*SLC8A1*	23	DHPR	*CACNA1C*, *CACNA1D**, *CACNA2D2*, *CACNB2**, *CACNB3*			
4	SGCG	*SGCG*	9	ACTG1	*ACTB**, *ACTG1*	14	MYH6/7	*MYH6**	19	Gs	*GNAS*	24	TNF-α	*TNF*			
5	SGCA	*SGCA*	10	Titin	*TTN*	15	MYBPC	*MYBPC3*	20	AC	*ADCY3, ADCY4 ADCY5, ADCY9*	25	TNF-β	*TGFB2*			

Figure S6: The BP-proteins in the KEGG-defined dilated cardiomyopathy (DCM) pathway and their PPI network. The BP proteins are shown (A) in the context of the pathway map, (B) with their gene symbols in respect to the numbered pathway map node symbols, and (C) in their subnetwork of the GWAS-RbSP protein interactome. BP-proteins in (C) are denoted by the number of the associated pathway map node. The figure is colour-coded and structured as described in the legend of Figure 10.

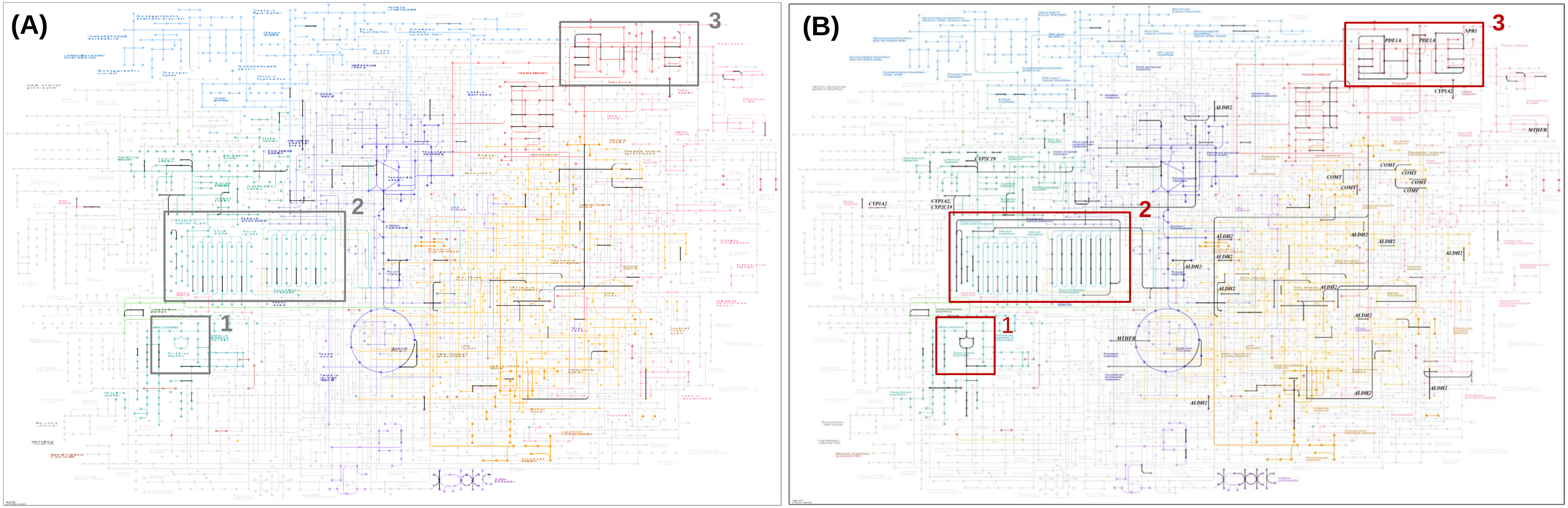

Figure S7: The metabolic reactions catalyzed by BP-GWAS proteins (A) and RbSP PPI network proteins (B). Both pictures have been exported from KEGG Pathway Mapper and the respective reactions are highlighted with black straight lines. Extending the protein set with the BN-GN shortest path intermediates increases the BP-enrichment of the Metabolic Pathways, revealing as BP-associated (1) steroid hormone synthesis, (2) lipid metabolism, (3) purine/pyrimidine biosynthesis, highlighted in red boxes. The gene symbols in (B) correspond to proteins targeted by anti-hypertensive drugs (Additional file 4).
